# Phytochemical Diversity and Multi-Endpoint In Vitro Biological Evaluation of *Sisymbrium irio* L.: Chemical Radical-Scavenging, Antibacterial, α-Amylase-/α-Glucosidase-Inhibitory, Cytotoxic, and Apoptosis-Associated Activities

**DOI:** 10.3390/cimb48100997

**Published:** 2026-09-27

**Authors:** Ibrahim M. Aziz, Rawan M. Alshalan, Adel A. Abdulmanea, Abdulaziz M. Almuqrin, Amal Khalaf Alghamdi

**Affiliations:** 1 Department of Botany and Microbiology, College of Science, King Saud University, P.O. Box 2455, Riyadh 11451, Saudi Arabia; ralshalaan@ksu.edu.sa (R.M.A.); aabdulmanea@ksu.edu.sa (A.A.A.); 2 Department of Clinical Laboratory Sciences, College of Applied Medical Sciences, King Saud University, Riyadh 12372, Saudi Arabia; aalmuqrin@ksu.edu.sa

**Keywords:** *Sisymbrium irio* L., natural products, phytochemicals, chemical radical-scavenging activity, antibacterial activity, α-amylase inhibition, α-glucosidase inhibition, cytotoxicity, apoptosis-associated gene expression

## Abstract

*Sisymbrium irio* L. (Brassicaceae) is an indigenous wild edible plant widely distributed in Saudi Arabia and the Mediterranean region and traditionally used in Unani and folk medicine to treat respiratory, digestive, and inflammatory ailments. However, its phytochemical diversity and organ-specific pharmacological potential remain insufficiently characterized. This study evaluated the phytochemical constituents; total phenolic and flavonoid contents; and in vitro antioxidant, antibacterial, cytotoxic/antiproliferative, apoptosis-associated and in vitro α-amylase- and α-glucosidase-inhibitory activities of methanolic leaf and stem extracts (SILEs and SISEs, respectively). GC–MS profiling putatively identified 82 compounds in SILE and 85 in SISE, revealing distinct organ-specific profiles. SISE showed higher FCRC and TFC (88.12 ± 1.15 and 45.18 ± 1.28 mg/g, respectively) and greater DPPH and ABTS^+^ radical-scavenging capacities. In contrast, SILE exhibited greater antibacterial activity, particularly against *Staphylococcus aureus* and *Enterococcus faecalis*, with MIC values as low as 6.25 µg/mL. Both extracts showed in vitro concentration-dependent cytotoxic/antiproliferative activity against MCF-7 and HepG2 cells, accompanied by changes in the expression of apoptosis-associated genes. These findings indicate apoptosis-associated transcriptional responses but do not establish functional apoptosis or tumor-selective/anticancer activity. SILE exhibited moderate in vitro α-amylase- and α-glucosidase-inhibitory activity (IC_50_ = 26.25 µg/mL (95% CI: 18.53–37.19 µg/mL) and 53.94 µg/mL (95% CI: 47.05–61.83 µg/mL), respectively). Overall, this study provides the first integrated comparison of SILE and SISE across multiple in vitro bioactivities, highlighting distinct organ-specific pharmacological potential that requires more investigation. These results do not prove therapeutic efficacy and should be viewed as first in vitro screening findings.

## 1. Introduction

*Sisymbrium irio* L. (London rocket), belonging to the Brassicaceae (Cruciferae) family, is an annual herb that is widely spread in the Mediterranean region, Asia, and North Africa [1]. Members of the Brassicaceae family include more than 360 genera and 4000 species. Economically valuable crops include cauliflower, cabbage, and broccoli [2]. Traditionally, *S. irio* L. has been used in the Unani and Ayurvedic systems of medicine to treat various ailments, such as respiratory diseases, digestive troubles, fever, rheumatism, and inflammatory diseases [3]. Seeds were used as an expectorant and febrifuge and to cure voice trouble, while aerial parts were used as an expectorant, as well as for detoxification of the liver and wound healing [1]. Traditional preparations typically involve the decoction or infusion of dried plant material in water or ethanol, which is then provided orally in quantities of 5–10 g per day to adults [3].

The Brassicaceae family represents a high-diversity family of secondary metabolites, such as glucosinolates, flavonoids, phenolic acids, sterols, and terpenoids. These secondary metabolites have considerable bioactivities, such as antioxidant, antibacterial, anticancer, and anti-inflammatory activities [4]. Previous phytochemical studies on *S. irio* L. have identified flavonoids (apigenin, luteolin, kaempferol, and their glycosides), glucosinolates, fatty acids (linolenic, oleic, and erucic acids), alkaloids, and phenolic compounds [5]. Ten flavonoids have been isolated from aerial parts, with the identification of apigenin-7-O-galactoside as a potent antioxidant molecule [5]. These bioactive chemicals have been shown to work through a variety of methods, including radical scavenging, enzyme inhibition, membrane disruption, and apoptotic pathway regulation. *S. irio* L. is one of the plants that have been recognized as part of the Saudi flora. Its area of natural occurrence includes the Arabian Peninsula, and it has been documented in herbaria as having been collected in Saudi Arabia [6]. It has also been reported as one of the dominant species in the arid regions of central Saudi Arabia in the Hail Province [7]. This makes it an important plant as a common weed in disturbed regions within the country and is thus a useful resource for locals. Previous studies have reported appreciable antioxidant activity in *S. irio* L. Methanolic extracts of the aerial parts showed relatively high total phenolic content (TPC) and notable antioxidant activity, with flavonoids suggested as potential contributors to the observed antioxidant capacity [8]. More recently, Chileh-Chelh et al. [9] reported that wild *S. irio* L. leaves contained higher phenolic and flavonoid contents than leaves grown under controlled conditions, accompanied by greater DPPH and ABTS radical-scavenging capacities. These findings suggest that antioxidant activity in *S. irio* L. may vary according to plant organ, growing conditions, and the composition of secondary metabolites.

In view of the phytochemically diverse nature of *S. irio* L., there is a need for a comprehensive analysis of its antioxidant, antibacterial, cytotoxic, and enzyme-inhibitory activities. Antioxidant activity can be assessed through radical scavenging tests, while assessment of antibacterial activity may be carried out against selected clinically relevant bacterial strains. The cytotoxic activity of the extracts against cell lines will serve as the basis for their possible antiproliferative activity, while enzyme inhibition assays will give some insight into their possible in vitro α-amylase-/α-glucosidase-inhibitory activities. Such in vitro tests are fundamental for the identification of promising activities before embarking on more complex and expensive in vivo tests.

For the above reasons, in vitro testing was chosen for initial screening of the activities of the extracts because it offers a rapid and inexpensive way to test for multiple biological activities with the use of standardized procedures. Animal cancer models were not included because they require substantial resources and extensive ethical approval. This work will focus on establishing an in vitro profile of the biological activities of the extracts of *S. irio* L.

Despite its documented ethnopharmacological importance, few comparative studies have examined the phytochemical composition and in vitro biological activities of different plant parts. The leaves and stems are two physiologically unique organs with separate biosynthetic capabilities; leaves function primarily in photosynthesis and contribute to phenolic biosynthesis, whereas stems primarily provide structural and transport functions and might contain other protective phytochemicals. Knowledge about these differences in physiological features of the organs is necessary for proper usage of active phytochemicals. It is important to remember that although the traditional *S. irio* L. preparation is usually either an aqueous or ethanolic decoction, absolute methanol was chosen for the purpose of this research due to its ability to extract various polar and semipolar active phytochemicals [10,11]. In the present study, methanol was selected specifically for analytical phytochemical extraction and preliminary in vitro screening rather than for direct biomedical or therapeutic application. For future food, pharmaceutical, or biomedical applications, ethanol, aqueous ethanol, or other application-appropriate extraction systems may provide more suitable alternatives.

Therefore, the present study aimed to determine and compare the chemical composition of methanolic *S. irio* L. leaf extract (SILE) and stem extract (SISE) using gas chromatography–mass spectrometry (GC–MS) analysis and to quantify their Folin–Ciocalteu reducing capacity (FCRC) and total flavonoid content (TFC). Furthermore, the study evaluated the antioxidant, antibacterial, cytotoxic, and in vitro α-amylase-/α-glucosidase-inhibitory activities of both extracts. These assays are exploratory in nature and do not aim to validate traditional antidiabetic or anticancer applications.

## 2. Materials and Methods

### 2.1. Plant Material Collection and Preparation of Extracts

*S. irio* L. leaves and stems were collected from naturally occurring plants growing in the garden of the College of Science, King Saud University, Riyadh, Saudi Arabia, during March 2026 in the spring season. The collection site was located at 24.7202016° N and 46.6182239° E. The plants were collected at the vegetative phenological stage and were growing under natural conditions within the garden environment. The plant material was authenticated by Prof. Dr. Mohammed Fasil, and a voucher specimen was deposited at King Saud University under voucher number KSU No. 16112 for taxonomic reference and future verification. Three naturally occurring *S. irio* L. plants from a single population were sampled. The leaves and stems collected from the three plants were pooled separately according to plant organ to obtain composite leaf and stem samples. The pooled materials were gently cleaned with distilled water and air-dried at room temperature (25 ± 2 °C) under good ventilation for 7 days. The dried leaves and stems were then separately ground into a fine powder.

The dried leaves and stems were separately extracted using 100% methanol under the conditions summarized in Table 1. Specifically, 10 g of powdered material was macerated in 100 mL of methanol for 48 h at room temperature (25 ± 2 °C) under continuous agitation. Methanol was selected because of its capacity to extract a broad range of bioactive phytochemicals, including phenolics, flavonoids, and other polar secondary metabolites [9]. The extraction was performed independently three times for each pooled plant-organ sample, using separate 10 g portions of the pooled powdered material. Because the plant materials from the three individual plants were pooled before extraction, these three extractions represent independent experimental/extraction replicates from a single composite biological sample rather than three independent biological replicates [10]. The extracts were filtered through Whatman No. 1 filter paper (Cytiva, Little Chalfont, Buckinghamshire, UK) and concentrated under reduced pressure using a rotary vacuum evaporator (BO410, Yamato Scientific Co., Ltd., Tokyo, Japan) at temperatures below 40 °C. The resulting dried extracts were stored at 4 °C until analysis. The extraction yield was calculated separately for each independent extraction using the following equation: Extraction yield (%) = (weight of dried extract/weight of dried plant material) × 100 [11].

### 2.2. GC–MS Analysis

The phytochemical composition of SILE and SISE was characterized by GC–MS on an Agilent Technologies system (Santa Clara, CA, USA) equipped with a split/splitless injector and a DB-5MS fused-silica capillary column (30 m × 0.25 mm i.d., 0.25 μm film thickness). Helium served as the carrier gas at a constant flow rate of 1.0 mL/min. The oven temperature program was set to 40 °C for 3 min, then increased to 280 °C at 7.5 °C/min and held for 5 min before being finally raised to 290 °C and maintained for 1 min. Samples (1 μL) were injected in split mode (1:10). Mass spectra were acquired in electron impact (EI) mode at 70 eV over an *m*/*z* range of 40–500. The ion source, interface, and quadrupole temperatures were maintained at 230 °C, 280 °C, and 150 °C, respectively. Compound assignments were based on spectral matching against the Wiley and National Institute of Standards and Technology (NIST) libraries, with similarity scores reported as supporting evidence for the putative or tentative annotations. Because no derivatization was performed before GC–MS analysis, highly polar and nonvolatile constituents may be under-represented or absent from the detected phytochemical profile. Compound identification levels were assigned as Level 2 (putative identification), Level 3 (tentative identification), or Level 4 (unknown compounds) according to metabolomics guidelines [12].

### 2.3. Determination of FCRC and TFC

The FCRC and TFC of SILE and SISE were determined using spectrophotometric colorimetric methods. For FCRC analysis, the Folin–Ciocalteu method described by Wolfe and Liu (2003) [13] was employed with slight modifications. Briefly, 0.1 mL of extract solution (1 mg/mL) was mixed with 3 mL of distilled water, followed by the addition of 2 mL of 20% (*w*/*v*) sodium carbonate (Na_2_CO_3_) solution. The reaction mixture was incubated in the dark at 25 °C for 30 min, and the absorbance was measured directly in cuvettes using a Thermo Scientific™ Multiskan SkyHigh Microplate Spectrophotometer (Cat. No. A51119700C; Thermo Fisher Scientific, Waltham, MA, USA). Gallic acid standard solutions (25–200 μg/mL) were used to construct the calibration curve. The FCRC was calculated using the calibration equation of *y* = 0.0081*x* + 0.0783, with a coefficient of determination (R^2^) of 0.9991. The results are expressed as milligrams of gallic acid equivalents per gram of dry extract (mg GAE/g dry extract).

For TFC determination, the aluminum chloride (AlCl_3_) colorimetric method described by Ordonez et al. (2006) [14] was used with minor modifications. Briefly, 500 μL of extract solution (2 mg/mL) was mixed with 1 mL of 2% (*w*/*v*) AlCl_3_ solution and 3 mL of sodium acetate solution (50 g/L). A volume of 50 microliters of acetic acid was added, and the mixture was incubated in the dark at 25 °C for 60 min. The absorbance was measured directly in a cuvette at 420 nm using a Thermo Scientific™ Multiskan SkyHigh Microplate Spectrophotometer (Cat. No. A51119700C; Thermo Fisher Scientific, Waltham, MA, USA). Quercetin standard solutions (25–200 μg/mL) were used to construct the calibration curve. The TFC was calculated using the calibration equation of *y* = 0.0018*x* + 0.117, with an R^2^ of 0.9995. The results are expressed as milligrams of quercetin equivalents per gram of dry extract (mg QE/g dry extract).

### 2.4. Antibacterial Activity

To evaluate the antibacterial activity of SILE and SISE, six bacterial strains were selected, comprising three Gram-positive bacteria—*Staphylococcus aureus* (ATCC 29213), *Enterococcus faecalis* (ATCC 29212), and *Bacillus subtilis* (ATCC 23857)—and three Gram-negative bacteria—*Escherichia coli* (ATCC 25922), *Klebsiella pneumoniae* (ATCC 13883), and *Pseudomonas aeruginosa* (ATCC 27853). The bacterial panel was selected to provide a balanced representation of Gram-positive and Gram-negative organisms and to include clinically relevant and widely used reference strains for preliminary antibacterial screening. The inclusion of both bacterial groups allowed for comparison of the susceptibility of the extracts across organisms with different cell-envelope characteristics. All strains were obtained from the American Type Culture Collection (ATCC), ensuring the use of standardized reference strains. Antibacterial activity was assessed using the agar well diffusion method as previously described in [15], with slight modifications. Briefly, bacterial inocula were standardized to 1.5 × 10^8^ colony-forming units (CFU)/mL, corresponding to a 0.5 McFarland turbidity standard, using a nephelometer (SN 070500012093, Becton, Dickinson and Company, Sparks, MD, USA). Using a sterile L-shaped spreader, 100 μL of each standardized bacterial suspension was evenly spread over the Mueller–Hinton agar (MHA, Oxoid, Basingstoke, UK) surface under aseptic conditions, and the plates were allowed to dry for approximately 30 min before the wells were prepared. Wells measuring 6 mm in diameter were aseptically bored into the agar using a sterile cork borer, ensuring uniform spacing between wells. SILE and SISE were prepared in 0.01% DMSO at four concentrations (100, 200, 400, and 800 μg/mL), and 100 μL of each solution was dispensed into the designated wells using a micropipette. Chloramphenicol (25 µg/mL) was used as the positive reference control for all six bacterial strains, whereas 0.01% DMSO served as the negative control (vehicle control). The plates were allowed to pre-diffuse at 4 °C for 30 min and were then incubated at 37 °C for 24 h. Antibacterial activity was assessed by measuring the diameter of the zones of inhibition (ZoIs) surrounding each well, expressed in millimeters (mm).

The minimum inhibitory concentration (MIC) and minimum bactericidal concentration (MBC) of SILE and SISE were determined by the broth microdilution method [16], using 2,3,5-triphenyl tetrazolium chloride (TTC) as a bacterial growth indicator. For the broth microdilution assay, 100 μL of Mueller–Hinton broth (MHB) containing two-fold serial dilutions of each extract (6.25–800 μg/mL) was dispensed into 96-well microplates, followed by the addition of 10 μL of standardized bacterial suspension (approximately 5 × 10^6^ CFU/mL), resulting in a final inoculum concentration of approximately 4.5 × 10^5^ CFU/mL in each well. After incubation at 37 °C for 24 h, 20 μL of TTC solution (2 mg/mL in phosphate-buffered saline [PBS]) was added to each well, and plates were incubated for an additional 20 min. To exclude possible chemical reduction of TTC or direct color interference by the extracts, extract-only control wells containing SILE or SISE at the tested concentrations but without bacterial inoculum were included and processed under the same conditions as the experimental wells. Wells exhibiting pink coloration were considered positive for bacterial growth, whereas colorless wells indicated inhibition of bacterial growth. For MBC determination, aliquots from wells showing no visible growth were sub-cultured onto fresh MHA plates and incubated at 37 °C for an additional 24 h. The MBC was defined as the lowest extract concentration at which no bacterial growth was observed following subculture onto extract-free MHA.

### 2.5. Evaluation of Chemical Radical-Scavenging Capacities

The chemical radical-scavenging capacities of SILE and SISE were assessed using DPPH (2,2-diphenyl-1-picrylhydrazyl) and ABTS^+^ [2,2′-azino-bis(3-ethylbenzothiazoline-6-sulfonic acid)] radical-scavenging assays following Tian et al. (2020) [17] and Yu et al. (2013) [18], respectively, with slight modifications. For the DPPH assay, extracts at concentrations of 0 (methanol, vehicle control), 100, 200, and 400 μg/mL were prepared in methanol and mixed with an equal volume of freshly prepared DPPH solution (0.08 mM). The reaction mixtures were vortexed and incubated in the dark at room temperature (25 °C) for 30 min. After incubation, the absorbance was measured directly in a cuvette at 734 nm using a Multiskan SkyHigh Microplate Spectrophotometer (Cat. No. A51119700C; Thermo Fisher Scientific, Waltham, MA, USA).

For the ABTS^+^ assay, ABTS^+^ radicals were generated by reacting 7 mM ABTS solution with 2.45 mM potassium persulfate (K_2_S_2_O_8_) and allowing the mixture to stand in the dark at room temperature for 12–16 h. Before analysis, the resulting solution was diluted with PBS to an absorbance of 0.70 ± 0.02 at 734 nm. Subsequently, 100 μL of each extract solution of 0 (methanol, vehicle control), 50, 100, and 200 μg/mL was mixed with 3 mL of the ABTS^+^ working solution and incubated at room temperature for 6 min before measuring the absorbance at 734 nm using a Thermo Scientific™ Multiskan SkyHigh Microplate Spectrophotometer (Cat. No. A51119700C; Thermo Fisher Scientific, Waltham, MA, USA). Ascorbic acid was used as the positive control.

For both DPPH and ABTS^+^ assays, ascorbic acid was included as the positive reference control. Extract-specific blanks were prepared to account for the intrinsic absorbance and turbidity of the extracts, and the corresponding blank values were subtracted before calculating radical-scavenging capacity.

The percentage radical-scavenging capacity was calculated using the following equation:

Scavenging capacities (%) = [(A_control_ − A_sample_)/A_control_] × 100

where A_control_ and A_sample_ represent the absorbance values of the control and sample, respectively. The half-maximal inhibitory concentration (IC_50_) values were calculated from concentration–response curves using nonlinear regression analysis in GraphPad Prism software (version 5.0; GraphPad Software Inc., La Jolla, CA, USA).

### 2.6. In Vitro Cytotoxic/Antiproliferative Activity

The cell lines employed in this study, human breast adenocarcinoma MCF-7 (ATCC HTB-22) and human hepatocellular carcinoma HepG2 (ATCC HB-8065), were obtained from the Virology Research Group (VRG), College of Science, King Saud University, Saudi Arabia. Prior to use, the cell cultures were tested for mycoplasma contamination using the LookOut^®^ Mycoplasma qPCR Detection Kit (Merck, Darmstadt, Germany) and were confirmed to be mycoplasma-free. Cells were used at passage 10 (MCF-7) and passage 12 (HepG2). The MTT (3-[4,5-dimethylthiazol-2-yl]-2,5-diphenyltetrazolium bromide) assay was used to assess the in vitro cytotoxic/antiproliferative effects of SILE and SISE against these established human cancer cell lines, which served as in vitro screening models for evaluating changes in cellular viability following treatment with the plant extracts [19,20]. The cell lines were cultivated under standard culture conditions (37 °C, 5% CO_2_, humidified atmosphere) in Dulbecco’s Modified Eagle Medium (DMEM; Gibco, Invitrogen, Grand Island, NY, USA) supplemented with 10% fetal bovine serum (FBS) and antibiotics (100 U/mL penicillin and 100 μg/mL streptomycin).

Cells were seeded into 96-well tissue culture plates at 1 × 10^4^ cells/well in 100 μL of complete medium and allowed to attach overnight. The next day, cells were treated with SILE or SISE at concentrations of 0 (0.01% DMSO, vehicle control), 25, 50, 100, and 200 μg/mL for 24 h. Cisplatin (30 μg/mL) was included as a positive reference control. Three replicate wells were used for each treatment concentration. After treatment, each well received 10 μL of MTT solution (5 mg/mL in PBS), and the plates were incubated at 37 °C for three hours. After carefully removing the culture media, 100 μL of DMSO was added to dissolve the purple formazan crystals. Absorbance at 570 nm was measured using an ELX-808 microplate (BioTek Laboratories, LLC, Shoreline, WA, USA). Cell-free MTT controls containing the plant extracts and MTT reagent (extract + MTT) but no cells were included as blanks to account for direct reduction of MTT and optical interference from plant constituents. The extracts’ impact on cell proliferation was evaluated by comparing the absorbance of treated and untreated cells. Cell viability (%) was calculated using the following equation:

Cell viability (%) = (Absorbance of treated cells/Absorbance of control cells) × 100

Dose–response curves were generated, and the IC_50_ values were calculated using nonlinear regression analysis in GraphPad Prism software (version 5.0; GraphPad Software Inc., La Jolla, CA, USA).

### 2.7. Quantitative Real-Time PCR Analysis of Apoptosis-Associated Gene Expression

To investigate apoptosis-associated transcriptional responses following treatment with SILE and SISE, MCF-7 and HepG2 cells were treated with their respective IC_50_ concentrations for 24 h. Total RNA was extracted using the RNeasy Micro Kit (Qiagen, Hilden, Germany), and cDNA was synthesized using the Wonder RT-cDNA Synthesis Kit (Euroclone, Milan, Italy). Gene expression was analyzed by RT-qPCR with SYBR Green Master Mix (Applied Biosystems, Foster City, CA, USA) on a 7500 Fast Real-Time PCR System (7500 Fast; Applied Biosystems, Foster City, CA, USA). The relative mRNA expression levels of the pro-apoptotic *caspase-3*, *caspase-8*, *caspase-9*, and *Bax* genes, as well as the anti-apoptotic *Bcl-2* and *Bcl-xL* genes, were evaluated. *GAPDH* was used as the housekeeping gene, and relative gene expression was calculated using the 2^−ΔΔCt^ method [21]. Results are expressed as fold changes relative to untreated control cells. Primer sequences used in this study are presented in Table 2. RT-qPCR experiments were performed using three independent biological replicates, with each biological replicate analyzed in triplicate technical PCR reactions. Technical replicates were averaged before statistical analysis.

### 2.8. In Vitro α-Amylase/α-Glucosidase Inhibitory Activity

The inhibitory effects of SILE and SISE on in vitro α-amylase and α-glucosidase activities were evaluated following previously described methods, with minor modifications [26,27]. The extracts were tested at concentrations of 0 (0.01% DMSO, vehicle control), 25, 50, 100, and 200 μg/mL. Acarbose (25–200 μg/mL) was used as the positive reference control, and 0.01% DMSO was included as the vehicle control.

For the in vitro α-amylase-inhibitory assay, porcine pancreatic α-amylase (Type VI-B; ≥5 U/mg solid; Sigma-Aldrich, St. Louis, MO, USA; Cat. No. A3176) was used at a final activity concentration of 2 U/mL. A volume of 200 μL of extract solution was mixed with 200 μL of α-amylase solution prepared in phosphate buffer (0.02 M Na_2_HPO_4_/NaH_2_PO_4_ containing 0.006 M NaCl, pH 6.9) and incubated at 37 °C for 10 min. Then, 200 μL of 1% soluble starch solution was added, and the reaction mixture was incubated at 37 °C for an additional 3 min. The reaction was terminated by adding 200 μL of DNSA reagent, followed by heating at 85 °C for 10 min. After cooling, the mixture was diluted with distilled water, and absorbance was measured at 540 nm using a Thermo Scientific Multiskan Sky spectrophotometer (Thermo Fisher Scientific, Waltham, MA, USA).

*Saccharomyces cerevisiae* α-glucosidase (1 U/mL) was prepared in 0.1 M phosphate buffer (pH 6.9) for the α-glucosidase-inhibitory test. *S. cerevisiae* α-glucosidase was used as an in vitro screening model for the preliminary assessment of α-glucosidase-inhibitory activity. Because this yeast enzyme does not directly represent mammalian intestinal α-glucosidase, the results were not interpreted as direct evidence of inhibition of human intestinal α-glucosidase. A reaction mixture comprising 50 μL of enzyme solution, 250 μL of phosphate buffer, and 100 μL of extract solution at concentrations of 0 (0.01% DMSO, vehicle control), 25, 50, 100, and 200 μg/mL was pre-incubated at 37 °C for 20 min. The reaction was then initiated by adding 10 μL of *p*-nitrophenyl-α-D-glucopyranoside (pNPG, 10 mM) and incubated at 37 °C for 30 min. The reaction was terminated by adding 650 μL of 1 M sodium carbonate. The release of *p*-nitrophenol was measured spectrophotometrically at 405 nm using a Thermo Scientific Multiskan Sky microplate reader. For both in vitro α-amylase and α-glucosidase assays, extract-specific blanks were prepared at each tested concentration by following the same experimental procedure but omitting the corresponding enzyme. These blanks were used to correct for intrinsic extract color, turbidity, reducing sugars, and other potential optical interferences with DNSA at 540 nm or *p*-nitrophenol at 405 nm. The absorbance of each extract blank was subtracted from the corresponding sample absorbance before calculating enzyme inhibition. The percentage inhibition was calculated according to the following equation:

Inhibition (%) = [(A_control_ − A_sample_)/A _contro_l] × 100

where A _control_ and A _sample_ represent the absorbance values of the negative control and sample, respectively. The IC_50_ values were determined from concentration-dependent inhibition curves using nonlinear regression analysis in GraphPad Prism software (version 5.0; GraphPad Software Inc., La Jolla, CA, USA).

### 2.9. Statistical Analysis

All experiments were performed using three independent extraction/experimental replicates obtained from pooled leaf or stem material. Thus, *n* = 3 represents independent extraction/experimental replicates from a single composite plant-organ sample and not three independent biological plant samples. Technical replicates were averaged before statistical analysis. Accordingly, statistical comparisons between SILE and SISE were interpreted as reflecting reproducibility within the respective pooled plant-organ samples and were not considered evidence of generalizable organ-specific differences in *S. irio* L. at the species level.

All statistical analyses were performed using GraphPad Prism software (version 5.0; GraphPad Software Inc., La Jolla, CA, USA). For experiments involving multiple experimental factors, factorial analysis of variance (ANOVA) was used. MTT data were analyzed using three-way ANOVA, whereas ZoI data were analyzed using three-way ANOVA, with extract, concentration, and bacterial species as factors. DPPH and ABTS^+^ data were analyzed using two-way ANOVA, with extract and concentration as factors. α-amylase and α-glucosidase inhibition data were analyzed using two-way ANOVA, with extract and concentration as factors. Significant effects were followed by Tukey’s multiple-comparison test where appropriate. Direct comparisons of FCRC and TFC between SILE and SISE were performed using an unpaired Welch’s *t*-test based on the three extraction/experimental replicates. A two-sided *p* < 0.05 was considered statistically significant. Statistical significance is reported using the predefined *p* < 0.05 threshold, as indicated in the corresponding Results and figure captions. Exact *p*-values and standardized effect-size estimates were not systematically calculated or reported for all analyses and, therefore, are not presented as a general reporting requirement.

IC_50_ values for DPPH, ABTS^+^, MTT, in vitro α-amylase, and α-glucosidase were estimated from concentration–response data by nonlinear regression using GraphPad Prism. The corresponding 95% confidence intervals (CIs) for IC_50_ estimates were obtained from the nonlinear regression models and are reported alongside the IC_50_ values in the Results and relevant figure captions. A four-parameter logistic (4PL) model with variable slope was used: Y = Bottom + (Top − Bottom)/[1 + 10^((LogIC^_50_^−X)×HillSlope)^], where *Y* is the measured response; *X* is the logarithm of the inhibitor concentration; Top and Bottom are the upper and lower asymptotic response plateaus, respectively; Log IC_50_ is the logarithm of the concentration producing 50% inhibition; and HillSlope is the slope factor. The IC_50_ was calculated as 10^Log IC^_50_. The four model parameters were estimated by nonlinear least-squares regression. The upper and lower asymptotes and Hill slope were estimated from the experimental data rather than fixed to predetermined values, unless otherwise specified by the assay. The zero-concentration vehicle–control values were used as the baseline experimental controls and were not treated as inhibitor concentrations in the logarithmic transformation. For each fitted concentration–response model, the residual degrees of freedom were calculated as the number of observations included in the fit minus the number of fitted parameters. Goodness of fit was assessed using the R^2^ and inspection of the residuals.

## 3. Results

### 3.1. Extraction Yields

Methanolic extraction of the plant material yielded 10.18 ± 2.28% (*w*/*w*) for SILE and 9.38 ± 1.27% (*w*/*w*) for SISE, based on three independent extractions and expressed as percentages of the initial dry weight.

### 3.2. Chemical Compositions of the SILE and SISE

GC–MS analysis revealed a rich and diverse phytochemical profile in both SILE and SISE extracts, with 82 and 85 compounds putatively identified, respectively, indicating slightly greater chemical diversity in the SISE. The chromatographic profiles exhibited several prominent peaks corresponding to major phytoconstituents, reflecting the chemical complexity of both extracts. Table 3 and Table 4 present detailed information on the putatively identified compounds, including retention time (Rt), peak area (%), molecular formula (MF), and molecular weight (Mw), while the corresponding chromatograms are shown in Figure 1 and Figure 2. In SILE, the most abundant compound was α-linolenic acid (15.67%), followed by n-hexadecanoic acid (6.22%), 4H-pyran-4-one, 2,3-dihydro-3,5-dihydroxy-6-methyl- (5.87%), 2-methoxy-4-vinylphenol (5.09%), phytol (4.95%) and 3′,5′-dimethoxyacetophenone (4.62%). In SISE, the major constituents were α-linolenic acid (12.12%), 4H-pyran-4-one, 2,3-dihydro-3,5-dihydroxy-6-methyl- (8.09%), β-sitosterol (7.17%), n-hexadecanoic acid (6.50%), α-linolenic acid, 2,3-dihydroxypropyl ester (5.87%), and 6,9,12,15-docosatetraenoic acid methyl ester (4.41%). Both extracts shared several major metabolites, including unsaturated fatty acids, phenolic compounds, and terpenoids; however, SILE was distinguished by its high abundance of cycloartane-type triterpenoids, whereas SISE contained higher levels of phytosterols and antioxidant-related compounds such as β-sitosterol, γ-sitosterol, and vitamin E. The predominance of fatty acids, terpenoids, sterols, tocopherols, and phenolic derivatives suggest that both extracts are rich sources of bioactive phytochemicals that may contribute to their antioxidant and pharmacological properties. The similarity values for each chemical compound are listed in Table 3 and Table 4.

### 3.3. FCRC and TFC

Quantitative analysis showed numerically higher FCRC and TFC values in SISE than in SILE. SISE exhibited an FCRC of 88.12 ± 1.15 mg GAE/g dry extract, compared with 85.00 ± 1.95 mg GAE/g dry extract for SILE; however, this difference was not statistically significant (*p* = 0.091). Similarly, TFC was 45.18 ± 1.28 mg QE/g dry extract for SISE and 43.34 ± 1.84 mg QE/g dry extract for SILE, with no statistically significant difference (*p* = 0.236).

### 3.4. Chemical Radical-Scavenging Capacities of SILE and SISE

The chemical radical-scavenging capacities of SILE and SISE were evaluated using DPPH and ABTS^+^ radical-scavenging assays. In the DPPH assay (Figure 3A), both extracts exhibited concentration-dependent radical-scavenging capacity, with inhibition increasing from the vehicle control (0, methanol) to 100, 200, and 400 µg/mL. At 400 µg/mL, SISE showed higher scavenging activity (83.93 ± 0.94%) than SILE (76.64 ± 3.60%), although both were lower than the positive control (96.99 ± 1.96%). The estimated IC_50_ values were 97.33 µg/mL (95% CI: 89.44–105.92) for SISE and 121.39 µg/mL (95% CI: 100.36–146.84) for SILE, compared with 68.74 µg/mL (95% CI: 60.93–77.55) for ascorbic acid. The lower IC_50_ value of SISE indicates greater DPPH radical-scavenging capacity than SILE under the tested conditions. The 4PL concentration–response models showed R^2^ = 1.000 for ascorbic acid and SILE and R^2^ = 0.99997 for SISE.

Similarly, the ABTS^+^ assay (Figure 3B) showed a concentration-dependent increase in radical-scavenging capacity from the vehicle control (0, methanol) to 50, 100, and 200 µg/mL. At 200 µg/mL, SISE exhibited higher scavenging capacity (82.27 ± 1.19%) than SILE (76.88 ± 2.15%), while the positive control showed 98.33 ± 1.56% inhibition. The estimated IC_50_ values were 65.73 µg/mL (95% CI: 63.46–68.08) for SISE, 83.32 µg/mL (95% CI: 75.13–92.40) for SILE, and 48.22 µg/mL (95% CI: 43.71–53.20) for ascorbic acid. The 4PL models showed R^2^ = 1.000 for all three ABTS^+^ concentration–response curves. Thus, SISE exhibited a lower IC_50_ and higher radical-scavenging activity than SILE in the ABTS^+^ assay. Overall, SISE showed greater chemical radical-scavenging capacity than SILE in both assays, as reflected by its higher inhibition percentages and lower IC_50_ values.

### 3.5. Antibacterial Effects of SILE and SISE

The antibacterial activities of SILE and SISE were evaluated against six bacterial strains using ZoI, MIC, and MBC assays (Table 5 and Table 6). Both extracts showed concentration-dependent antibacterial activity relative to the 0.01% DMSO vehicle control. SILE generally exhibited greater antibacterial activity than SISE across the tested microorganisms, as reflected by larger ZoI values and lower MIC and MBC endpoint concentrations.

For SILE, the largest ZoI values at 800 µg/mL were observed against *S. aureus* (20 ± 2.57 mm) and *E. faecalis* (21 ± 2.11 mm), with MIC/MBC endpoint concentrations of 6.25/12.50 µg/mL and 6.25/12.50 µg/mL, respectively. *B. subtilis* also showed relatively high susceptibility, with MIC and MBC endpoint concentrations of 12.50 and 25.00 µg/mL, respectively. Among the Gram-negative bacteria, *E. coli* showed MIC and MBC endpoint concentrations of 50 and 100 µg/mL, respectively, whereas *K. pneumoniae* and *P. aeruginosa* showed MIC values of 25 µg/mL and MBC values of 50 µg/mL.

SISE also inhibited all tested bacterial strains but generally showed lower activity than SILE. Its greatest activity was observed against *S. aureus*, *E. faecalis*, and *B. subtilis*, whereas *E. coli* and *P. aeruginosa* were among the less susceptible strains, with MIC values of 50 µg/mL and MBC values of 100 µg/mL.

Overall, the extract treatments demonstrated measurable antibacterial activity relative to the vehicle control, with SILE showing stronger activity than SISE, particularly against the Gram-positive strains. Chloramphenicol was retained as a positive reference control for benchmarking of antibacterial potency rather than as the basis for determining whether the extracts produced a treatment effect.

### 3.6. In Vitro Cytotoxic/Antiproliferative Activity and Apoptotic Activities of SILE and SISE

The cytotoxic/antiproliferative effects of SILE and SISE against MCF-7 and HepG2 cells were evaluated using the MTT assay at concentrations of 0 (0.01% DMSO, vehicle control), 25, 50, 100, and 200 μg/mL for 24 h. Cisplatin (30 μg/mL) was included as a positive reference control (Figure 4A,B). Both extracts showed concentration-dependent cytotoxic/antiproliferative activity, resulting in a progressive reduction in cell viability. For SILE, cell viability decreased from 80.84 ± 1.85% to 25.24 ± 2.45% in MCF-7 cells and from 72.82 ± 0.95% to 20.98 ± 1.25% in HepG2 cells as the concentration increased from 25 to 200 μg/mL. The estimated IC_50_ values were 46.25 μg/mL (95% CI: 44.81–47.70) for MCF-7 and 42.69 μg/mL (95% CI: 41.49–43.89) for HepG2 cells. The corresponding 4PL concentration–response models showed R^2^ = 0.9998 and 1.0000, respectively. Similarly, SISE reduced cell viability from 89.84 ± 1.14% to 31.24 ± 1.14% in MCF-7 cells and from 85.18 ± 0.95% to 24.43 ± 1.14% in HepG2 cells. The estimated IC_50_ values were 50.36 μg/mL (95% CI: 49.49–51.23) for MCF-7 and 42.73 μg/mL (95% CI: 42.03–43.43) for HepG2 cells, with corresponding R^2^ values of 0.9949 and 0.9966, respectively. Thus, under the tested conditions, the IC_50_ values were lower in HepG2 cells than in MCF-7 cells for both extracts, indicating greater sensitivity of HepG2 cells to the tested extracts. Overall, both extracts exhibited notable in vitro cytotoxic/antiproliferative activity, with HepG2 cells showing greater sensitivity than MCF-7 cells.

To investigate apoptosis-associated transcriptional responses, MCF-7 and HepG2 cells were treated with SILE or SISE at their respective IC_50_ concentrations for 24 h, as shown in Figure 5A and B, respectively. Cisplatin (30 μg/mL) was used as a reference treatment (Figure 5C). Cells treated with 0.01% DMSO served as the vehicle control. Both extracts altered the transcriptional profile of apoptosis-associated genes in MCF-7 and HepG2 cells, including increased expression of pro-apoptotic markers and decreased expression of anti-apoptotic markers. In MCF-7 cells, SILE increased caspase-3, caspase-8, caspase-9, and Bax expression by 4.30-, 3.50-, 4.18-, and 3.19-fold, respectively, while Bcl-2 and Bcl-xL decreased to 0.40- and 0.50-fold, respectively. In HepG2 cells, SILE increased caspase-3, caspase-8, caspase-9, and Bax expression by 4.54-, 4.62-, 5.40-, and 3.55-fold, respectively, while Bcl-2 and Bcl-xL decreased to 0.39- and 0.45-fold. SISE produced a comparable apoptosis-associated transcriptional pattern in both cell models. These findings indicate changes in apoptosis-associated gene transcription but do not establish functional apoptosis. In particular, the reported increase in caspase-3 mRNA in MCF-7 cells should be interpreted cautiously because the present study did not include independent amplicon validation or the full set of RT-qPCR quality-control parameters described above. Therefore, this caspase-3 finding is considered preliminary and is not interpreted as evidence of activation of a caspase-3-dependent apoptotic pathway.

### 3.7. In Vitro α-Amylase- and α-Glucosidase-Inhibitory Activities

The in vitro α-amylase-/α-glucosidase-inhibitory activity of SILE and SISE was evaluated by measuring inhibition of in vitro α-amylase and α-glucosidase, using acarbose as the positive reference control. Both extracts showed concentration-dependent inhibition, with activity increasing significantly from 25 to 200 µg/mL. However, the positive reference control (acarbose) showed the highest activity in both assays.

In the in vitro α-amylase assay (Figure 6A), SILE produced inhibition ranging from 25.84 ± 1.83% to 80.64 ± 1.36%, with an IC_50_ of 26.25 µg/mL (95% CI: 18.53–37.19 µg/mL), whereas SISE showed lower inhibition (22.17 ± 1.47% to 73.49 ± 1.98%) and a higher IC_50_ of 41.91 µg/mL (95% CI: 34.21–51.33 µg/mL). The positive reference control (acarbose) exhibited the strongest activity, with an IC_50_ of 20.40 µg/mL (95% CI: 16.18–25.72 µg/mL). The corresponding 4PL models yielded R^2^ values of 1.0000 for SILE, 0.9997 for SISE, and 0.9790 for acarbose.

Similarly, in the in vitro α-glucosidase assay (Figure 6B), SILE showed greater inhibition (17.45 ± 1.43% to 82.64 ± 1.36%) and a lower IC_50_ (53.94 µg/mL (95% CI: 47.05–61.83 µg/mL) than SISE (13.17 ± 0.99% to 75.49 ± 1.94%; IC_50_ = 78.06 µg/mL (95% CI: 68.44–89.02 µg/mL). The positive control remained the most potent inhibitor, with an IC_50_ of 25.51 µg/mL (95% CI: 19.92–32.67 µg/mL). The corresponding 4PL models yielded R^2^ values of 0.9988 for SILE, 0.9847 for SISE, and 1.0000 for acarbose.

Overall, as shown in Figure 6A,B, SILE consistently exhibited stronger in vitro α-amylase/α-glucosidase inhibitory activity than SISE, as evidenced by higher inhibition percentages and lower IC_50_ values, although both extracts were less potent than the positive reference control (acarbose).

## 4. Discussion

The extraction yields were 10.18 ± 2.28% (*w*/*w*) for SILE and 9.38 ± 1.27% (*w*/*w*) for SISE. In general, SILE contains higher amounts of primary and secondary metabolites than SISE. Leaves contain a wide variety of biosynthesized polar secondary metabolites and are therefore easily extracted by methanol. Conversely, the main components of plant stems include non-extractable structures (lignified cellulose, hemicellulose, and structural polysaccharides) [28]. Studies evaluating the phytochemical composition of Brassicaceae plants have generally shown that leaf extracts contain higher levels of bioactive compounds than stem extracts [29,30,31]. Notably, the extraction yield values for the investigated plants are similar to those reported for other species of the *Sisymbrium* genus and for other edible wild species of the Brassicaceae family [9,30,31,32]. The above information demonstrates that methanol is an adequate solvent for extracting a wide range of polar and moderately polar phytochemicals from the investigated plant materials.

The GC–MS analysis of the extracts showed an abundant and diverse phytochemical profile in the two samples, with 82 and 85 compounds putatively identified in the SILE and SISE samples, respectively, based on library matching, which require confirmation using authentic standards and retention indices. It should also be noted that GC–MS peak area percentages reflect relative abundance rather than absolute concentrations, as no internal standard or response parameters were used for measurement. These results indicate multiple important peaks for key phytoconstituents in the samples, highlighting the chemical diversity and complexity of the samples. The most common constituent in both samples is α-linolenic acid, an important omega-3 fatty acid and a major component of plant lipids, especially in leaf chloroplasts [33]. The presence of n-hexadecanoic acid (palmitic acid) at 6.22% in SILE and 6.50% in SISE highlights the wide distribution of saturated fatty acids throughout the plant organs. Importantly, the two extracts had distinctive metabolic profiles, reflecting their different physiological functions. The SILE sample is distinguished by the abundance of α-linolenic acid (15.67%), as well as high quantities of 2-methoxy-4-vinylphenol (5.09%) and phytol (4.95%). Triterpenoids such as squalene and oxidosqualene are biosynthetic precursors of sterols and other triterpenoids. They are essential constituents of cell membrane structures, helping maintain the stability and stress resistance of such membranes in photosynthesizing leaf structures exposed to changing environments [34]. Squalene (1.26% in SILE and 0.43% in SISE) and lupeol (0.81% in leaves) were putatively identified in the extracts, indicating the occurrence of triterpenoid-related metabolites. In contrast, SISE is rich in structure-retaining and protective phytosterols: β-sitosterol (7.17%), γ-sitosterol (2.07%), and tocopherol (vitamin E, 2.19%). The relative abundances of β-sitosterol and γ-sitosterol in stems may contribute to membrane stability in vascular and supportive tissues [35,36]. The n-hexane extract from the aerial parts of *S. irio* L. is abundant in β-sitosterol and its glycoside, which exhibit strong cytotoxic and antibacterial activity [37]. Squalene (1.26% in leaves and 0.43% in stems) and lupeol (0.81% in leaves) in both extracts are consistent with the presence of triterpenoid-related metabolites.

Earlier studies on *S. irio* L. have revealed the presence of α-linolenic acid, linoleic acid, and n-hexadecanoic acid in above-ground parts. In an earlier study, it was found that the seed oil of *S. irio* L. is rich in linolenic and palmitic acid methyl esters, at 38% and 6%, respectively [28]. Hailu et al. (2021) reported that different extracts of the seeds of *S. irio* L. are rich in linolenic, linoleic, oleic, cis-11-eicosanoic, erucic, and n-hexadecanoic acids, as well as other compounds, such as -tocopherol, cholesterol, n-hexadecanoic acid, and β-sitosterol [38]. Akhtar et al. (2020) reported that the shoot extract of Pakistani *S. irio* L. was rich in β-sitosterol, tert-butyldimethylsilyl ester, diisooctyl phthalate, 10-octadecenoic acid methyl ester, di-n-octyl phthalate, and 1,3-benzenedicarboxylic acid bis(2-ethylhexyl) ester [39]. Bagheri et al. (2019) used GC–MS analysis of the essential oils from Iranian *S. irio* L. seeds and leaves and reported that the seeds were rich in β-pinene, bicycloelemene, menthol, isopropyl isothiocyanate, and pulegone, whereas the leaves were rich in β-pinene, menthol, sabinene, and isobutyl isothiocyanate [40]. Another study from Saudi Arabia used GC–MS analysis of the methanolic extract of the aerial parts of *S. irio* L., which revealed a chemical profile of carbohydrates, sterols, triterpenes, coumarins, tannins, flavonoids, alkaloids, and saponins [5].

The variety of substances identified by GC–MS analysis is consistent with information on other plants in the Brassicaceae family. Plants of the *Sisymbrium* genus, which includes *S. irio* L., are known for seeds rich in polyphenols, glucosinolates, and glucosinolate metabolites with numerous biological activities [41]. The presence of 4H-pyran-4-one, 2,3-dihydro-3,5-dihydroxy-6-methyl (5.87% in SILE and 8.09% in SISE) indicates the presence of pyranone derivatives. However, this compound is not a phenolic substance, and its presence should not be interpreted as evidence of high phenolic content. The relatively higher levels of phenolic compounds and vitamin E detected in the stems may contribute to their antioxidant capacity; however, the physiological basis and functional significance of their accumulation require further investigation. Other plants of the Brassicaceae family are also rich in polyphenols; for example, *Coronopus didymus* (lesser swine cress) contains chlorogenic acid, kaempferol, ferulic acid, and quercetin [42]. Accounts report the presence of glucosinolates in the leaves of *S. irio* L., which is typical of plants of the Brassicaceae family, while apigetrin is the major phenolic compound [43]. A close relative of *S. irio* L. is *Sisymbrium officinale*, also known as hedge mustard, which contains glucosinolates and isothiocyanate hydrolysis products, explaining its use in aphonia treatment [44]. In addition, the Korean cabbage (*Brassica campestris* L. ssp. *Pekinensis*) has been reported to be highly rich in secondary metabolites, including carotenoids, sterols, phenolic acids, alkaloids, and glucosinolates [45]. Brindisi et al. (2023) determined new glucosinolates in the baby leaves of *Eruca vesicaria* subsp. sativa, *Brassica juncea*, and *B. oleracea* var. *capitata* L. (Brassicaceae) by GC/MS and identified Glucobarbarin, Glucoalyssin, glucocheirolin, glucoiberin, gluconapin, glucobrassicanapin, glucotropaeolin, and neoglucobrassicin [46].

SISE showed slightly higher FCRC and TFC than SILE, although these differences were not statistically significant and should not be interpreted as definitive organ-specific differences. The higher numerical values in SISE may reflect variation in the accumulation and distribution of reactive constituents between plant organs. Notably, the Folin–Ciocalteu assay measures overall reducing capacity, whereas the AlCl_3_ assay provides a colorimetric estimate of flavonoids. Although leaves are generally considered important sites of phenolic biosynthesis, stems may also accumulate phenolic compounds in their epidermal and cortical tissues as part of their protective functions against environmental and biotic stresses [47]. However, the present findings are descriptive and do not establish a specific physiological mechanism underlying the observed differences. Previous research on *S. irio* L. revealed the presence of phenols, flavonoids, saponins, and terpenoids in both leaf and stem tissues [8]. A previous study profiling the flavonoids in the aerial parts of *S. irio* L. identified eleven flavonoids, including apigenin, apigenin-7-galactoside, luteolin-7-O-glucoside, and kaempferol. Apigenin-7-galactoside was shown to be the most efficient antioxidant flavonoid, underscoring the significant contribution of these compounds to the plant’s bioactivity [5].

The high levels of phenolic and flavonoid components in the SILE and SISE samples are consistent with findings in other Brassicaceae species. For instance, Italian *B. oleracea* contained TFC of 14.50 ± 1.29 mg QE/g DW, total glucosinolates of 43.70 ± 1.09 mg SnE/g DW, and vitamin C of 7.21 ± 0.13 mg AA/g DW [48]. Tunisian *B. tournefortii* extracts included TPC of 9.9–21.78 mg/g, TFC of 2.36–6.6 mg/g, and 5% saponins, with the acetone extract showing the highest values [49]. Moreover, the ethanolic leaf extract of Italian *E. virgatum* subsp. virgatum had a high phenolic content of kaempferol derivatives compared with the water extract [50]. Flavonoids such as quercetin, kaempferol, and isorhamnetin, as well as hydroxycinnamic acids, are characteristic of *B. oleracea*, *B. rapa*, *B. napus*, and *B. juncea* and are known to be rich in these compounds [51]. However, previous research by Shukla et al. (2024) investigating the influence of low temperature on the chemical composition of *Brassicaceae* vegetables reported lower TPC (9.56 μg/mg) and TFC (14.48 μg/mg) values than those obtained in the present study [52]. This difference may be associated with variations in environmental conditions, particularly temperature, as well as differences in plant species, growth conditions, and developmental stage. The relatively high temperatures prevailing in Saudi Arabia may contribute to enhanced accumulation of phenolic compounds in *S. irio* L.; however, further studies are required to confirm the specific contribution of temperature to these variations.

Both extracts demonstrated concentration-dependent DPPH and ABTS^+^ radical-scavenging capacity, with SISE showing lower IC_50_ values and higher activity than SILE in both assays. These findings indicate greater in vitro chemical radical-scavenging capacity of SISE under the assay conditions. The higher FCRC, together with the presence of phenolic derivatives and other antioxidant-related constituents identified by GC–MS, may contribute to the observed radical-scavenging capacity. The higher radical-scavenging ability of SISE may be attributed to its slightly higher total phenolics and lipophilic antioxidants such as tocopherols and β-sitosterol [53]. These interpretations remain hypothetical and require validation through bioassay-guided fractionation and isolation of active compounds. Causality between the amount of each compound and antioxidant activity could be determined using correlation analysis and fractionation based on bioassay. Such studies would help clarify the contributions of individual phytochemicals to the observed biological activities. Previous research indicated that flavonoids isolated from *S. irio* L. exhibited reasonable antioxidant activity, particularly apigenin-7-O-galactoside [5]. Some phenolic acids, such as sinapic acid and ferulic acid derivatives, have been shown to possess radical-scavenging ability [54]. One notable finding in the current study is that the antioxidant activity of SISE exceeded that of SILE. This may be explained by the presence of lipophilic antioxidants, such as tocopherols and sterols, identified in the phytochemical analysis, which are commonly accumulated in vascular tissues to protect against lipid peroxidation during nutrient and water shortages, especially in arid, hot areas [55]. Similar findings were previously reported for the dose-dependent antioxidant activities of *S. irio* L. and related Brassicaceae species. The results on the leaf extract of wild *S. irio* L. obtained by Chileh-Chelh et al. (2025) demonstrated equivalent or better radical-scavenging activity (DPPH: 8.03–8.67 mmol TE/100 g DW; ABTS: 6.49–6.81 mmol TE/100 g DW) compared with that obtained under controlled cultivation [9]. Nengroo and Rauf (2019) analyzed the antioxidant activities of petroleum seed extracts from five medicinal temperate plants, including *S. irio* L., and demonstrated a dose-dependent increase in activity up to 83% at 200 μg/mL [56]. The enhanced activity of SISE corresponds to increased phenolic content, as in *Coronopus didymus*, where methanol and ethanol extracts exhibited higher DPPH-scavenging activity than the acetone extract [42]. Vitamin E, abundant in stems, increases radical-scavenging activity because tocopherols act as powerful lipophilic antioxidants [57]. Antioxidant activities of plants of the genus *Brassica* are attributed to polyphenols and glucosinolates [58]. Relatives of *Sisymbrium*, especially *S. officinale* [59], have also been reported as antioxidants. These findings underscore the need to examine different plant organs independently, as stems can be a good source of natural antioxidants and other bioactive compounds.

The antibacterial activity of SILE and SISE was evaluated against six bacterial strains representing both Gram-positive and Gram-negative organisms. Overall, SILE exhibited greater antibacterial activity than SISE across the tested strains. *S. aureus* and *E. faecalis* were particularly susceptible to SILE, showing ZoIs of 20 ± 2.57 and 21 ± 2.11 mm, respectively, at 800 µg/mL, with corresponding MIC/MBC endpoint concentrations ranging from 6.25 to 12.50 µg/mL. In contrast, the Gram-negative bacteria generally showed lower susceptibility. For example, *E. coli* exhibited MIC and MBC values of 50 and 100 µg/mL, respectively. The MIC and MBC endpoint concentrations further supported the observed antibacterial activity, with SILE generally showing lower endpoint concentrations than SISE. The observed difference in susceptibility between Gram-positive and Gram-negative bacteria may also partly reflect differences in bacterial envelope architecture, particularly the additional outer membrane present in Gram-negative bacteria [60,61]. The high antibacterial activity of the leaf extract may be attributed to its specific phytochemical profile, which includes high contents of phenolic compounds like 2-methoxy-4-vinylphenol (5.09%) and eugenol (0.29%). Nevertheless, the unique contributions of specific compounds to the antibacterial efficacy shown by the extract need further investigation via bioassay-directed fractionation. SILE contained a relatively high proportion of cycloartane triterpenoids (12.95%), which were absent in SISE, as well as volatile phenolic compounds such as 2-methoxy-4-vinylphenol (5.09%) and eugenol (0.29%). These compounds have been reported to affect bacterial cell membrane integrity and inhibit bacterial enzymes [62,63]. However, their specific contribution to the antibacterial activity observed in SILE cannot be established from the present data.

Previous studies have reported high antibacterial activity in solvent extracts of the aerial parts of *S. irio* L., particularly the n-hexane and ethyl acetate extracts, which inhibit the growth of *S. pyogenes*, *C. perfringens*, and *S. enteritidis* [64]. Another study showed that the choice of solvent for extracting *S. irio* L. seeds affects bacterial strain sensitivity, with ethanolic and methanolic preparations showing significant activity against *E. coli*, *S. aureus*, and *P. aeruginosa*, unlike water, acetone, and n-Hexane extracts [65]. Molecular docking studies have identified phytochemicals from *S. irio* L. seeds as efficient inhibitors of *S. aureus* dihydrofolate reductase [66], and substances such as “benzene-1,2-dicarboxylic acid, monoamide, N-(1-cyano-1-methylethyl)” as efficient DNA gyrase subunit B inhibitora [65]. The greater activity of SILE toward Gram-positive bacteria is supported by findings reported for the *Brassica* genus; seed extracts exert antibacterial activity against different strains [41]. Notably, the greater susceptibility of Gram-positive bacteria may partly reflect differences in cell-envelope structure, particularly the absence of an outer membrane [67]. Although the β-sitosterol content in the stem was relatively high (7.17% relative peak area), it was apparently insufficient to overcome the increased number of antibacterial phytochemicals in the leaves. Another species of the same family, *S. officinale*, has been studied for its bioactive compounds, including glucosinolates that provide antibacterial isothiocyanates [32]. Taken together, these findings may explain the observed antibacterial profile of *S. irio* L. extracts.

The MTT assay was used to assess the cytotoxic/antiproliferative effects of SILE and SISE on MCF-7 and HepG2 cells. Both extracts produced concentration-dependent reductions in cell viability. For SILE, the estimated IC_50_ values were 46.25 µg/mL (95% CI: 44.81–47.70) in MCF-7 cells and 42.69 µg/mL (95% CI: 41.49–43.89) in HepG2 cells. For SISE, the corresponding IC_50_ values were 50.36 µg/mL (95% CI: 49.49–51.23) and 42.73 µg/mL (95% CI: 42.03–43.43). These findings indicate concentration-dependent in vitro cytotoxic/antiproliferative activity under the tested conditions but do not establish cancer selectivity or a therapeutic anticancer effect.

To explore the possible mechanisms underlying the observed cytotoxicity, the expression of apoptosis-associated genes was assessed. Both SILE and SISE increased the mRNA expression of *caspase-3*, *caspase-8*, *caspase-9*, and *Bax* and decreased *Bcl-2* and *Bcl-xL* expression in MCF-7 and HepG2 cells. These transcriptional changes are consistent with an apoptosis-associated response; however, they do not demonstrate activation of the apoptotic pathway or functional apoptosis because caspase activity, proteolytic cleavage of caspases or poly (ADP-ribose) polymerase (PARP), and protein-level changes were not assessed. SILE and SISE upregulated the expression of mRNA of caspase-3, caspase-8, caspase-9, and Bax while downregulating the expression of Bcl-2 and Bcl-xL mRNA. These transcriptional changes do not establish functional apoptosis but support an apoptosis-associated transcriptional response. In order for caspases to be active and induce apoptosis, their proteolytic modification is needed, which was not investigated in the present study. Greater sensitivity of HepG2 cells to both extracts might be attributed to the inherent characteristics of cellular metabolism, apoptosis, or drug efflux in these two cell lines. This difference in sensitivity was not explored in detail in the present study and requires further investigation. The relatively strong activity of SISE against HepG2 cells may be related to its relatively high β-sitosterol content; however, this association requires experimental validation [68]. It is important to mention that MCF-7 cells lack caspase-3 activity because of the 47-base-pair deletion in the *caspase-3* gene. Thus, the increased expression of CASP3 mRNA in MCF-7 cells needs to be treated with caution. The available information does not allow one to assume that the caspase-3-dependent pathway is responsible for the noted cytotoxicity. In addition, MCF-7 cells are able to die in a caspase-3-independent manner.

A previous study reported that the n-hexane fraction of *S. irio* L. aerial parts exhibited cytotoxic activity against MCF-7, HCT-116, and HepG2 cancer cells, with IC_50_ values between 11.7 µg/mL and 13.4 µg/mL [64]. The dichloromethane fraction of wild *S. irio* L. was highly active against HCT-116 cells (IC_50_ = 5.42 µg/mL), whereas the ethanol extract showed a mild to moderate antiproliferative effect against HT-29 human colorectal cancer cells (IC_50_ = 210–380 µg/mL) after 72 h of treatment [9]. Additionally, ZnO nanoparticles biosynthesized from different *S. irio* L. extracts exhibited strong anticancer activity against BHK (Baby hamster kidney cell line) and HepG2 (hepatocellular carcinoma cell line), reaching 90% with an IC_50_ = 0.37–0.48 mg/mL [69]. The Brassicaceae family is well known for its anticancer activity, mediated by isothiocyanates and indoles that induce apoptosis and inhibit cell proliferation [70,71]. A previous study showed that 6 µM sulforaphane, which is well expressed in different Brassicaceae species, induced potent anti-leukemic activity [72]. *B. oleracea* extract induced anticancer activity against HT-29 cells (IC_50_ = 60.8 µg/mL) and markedly upregulated several genes in the caspase-dependent apoptotic pathway, including *caspase-9*, *mapk10*, *mapk11*, *fas*, and *kat2b* [73].

SILE and SISE were assessed for their in vitro inhibitory effects on α-amylase and α-glucosidase activities. Both extracts showed concentration-dependent inhibition, with inhibition increasing from 25 to 200 µg/mL. SILE exhibited greater inhibitory activity against both enzymes than SISE, as indicated by higher inhibition percentages and lower IC_50_ values. For in vitro α-amylase, the IC_50_ of SILE was 26.25 µg/mL (95% CI: 18.53–37.19 µg/mL), whereas that of SISE was 41.91 µg/mL (95% CI: 34.21–51.33 µg/mL). For α-glucosidase, the IC_50_ of SILE was 53.94 µg/mL (95% CI: 47.05–61.83 µg/mL), whereas that of SISE was 78.06 µg/mL (95% CI: 68.44–89.02 µg/mL). This higher efficiency of the in vitro α-amylase-/α-glucosidase-inhibitory activity of the SILE is probably due to the higher quantities of cycloartane-type triterpenoids and certain phenolic compounds, which have already proven their ability to interact with carbohydrate-hydrolyzing enzymes through hydrogen bonds and hydrophobic interactions [74]. In an investigation of seven wild edible Palestinian plants, it was found that the lipophilic fractions of *S. irio* L. leaves possessed the highest porcine pancreatic α-amylase-inhibitory effect, equal to the IC_50_ value of 7.72 µg/mL. This finding aligns with our observation that the leaf-derived SILE, containing lipophilic triterpenoids and fatty acids, demonstrates better enzyme-inhibiting activity compared with the stem extract. Moreover, because the α-glucosidase assay employed *S. cerevisiae* α-glucosidase, the present findings should not be directly extrapolated to mammalian or human intestinal α-glucosidase inhibition. Further studies using mammalian or human intestinal enzyme systems are required to establish the physiological relevance of the observed enzyme-inhibitory effects.

*B. oleracea* var. showed inhibitory effects against α-amylase and α-glucosidase enzymes, with IC_50_ values of 3.08 mg/mL and 22.63 mg/mL, whereas in silico analysis revealed an antidiabetic pathway through castasterone and 26-hydroxybrassinolide phytochemicals [75]. *Coronopus didymus* extracts also significantly inhibit α-glucosidase, with ethanol extracts showing IC_50_ values of 0.41 mg/mL and even exceeding the activity of acarbose at certain doses [42]. On the other hand, another study reported potential antihyperglycemic activity of *B. oleracea* leaves (300 mg/kg) in alloxan-induced diabetic rats through the accumulation of quercetin and kaempferol due to the putative role of an ATP-binding cassette (ABC) transporter [76]. In the seed and flower extracts of *B. incana*, an inhibitory effect against α-glucosidase (IC_50_ = 0.968 and 1.921 mg/mL) was reported [77]. The observed in vitro α-amylase-/α-glucosidase-inhibitory activity in both extracts may be attributed to phenolic compounds, such as sinapic acid and ferulic acid derivatives, which can form hydrogen bonds with enzyme active sites and inhibit α-amylase and α-glucosidase [78,79].

The current research provides novel findings regarding *S. irio* L. This research is the first to fully examine the antioxidant, antibacterial, in vitro cytotoxic/antiproliferative, and in vitro α-amylase-/α-glucosidase-inhibitory properties of SILE and SISE within a single experimental design. Nevertheless, it must be stressed that this is a multi-endpoint screening strategy but not an example of a multi-target drug development approach. Such an evaluation of the bioactivities of the substances allows for the detection of promising directions for further study. Whereas separate pharmacological activities have been documented previously, the simultaneous assessment of multiple bioactivities in both leaf and stem tissues enables a comprehensive comparison of their phytochemical and in vitro biological profiles and facilitates the identification of differences between plant organs. To the best of our knowledge, this is the first report showing that SILE and SISE induce transcriptional changes associated with apoptosis-related signaling in MCF-7 and HepG2 cells. Treatment increased the mRNA expression of *caspase-8*, *caspase-9*, and *Bax* while decreasing *Bcl-2* and *Bcl-xL* expression, suggesting the involvement of apoptosis-related mechanisms in the observed cytotoxic/antiproliferative effects. The findings highlight the in vitro α-amylase- and α-glucosidase-inhibitory potential of *S. irio* L., with SILE exhibiting the stronger effect, and contribute to understanding of its relationship with the plant’s phytochemical composition.

Several limitations should be acknowledged when interpreting the present findings. The observed chemical radical-scavenging capacity and cellular cytotoxicity represent distinct experimental endpoints and should not be considered mutually exclusive. DPPH and ABTS^+^ assays measure the ability of extract constituents to interact with chemically generated radicals under cell-free conditions, whereas the MTT assay evaluates cellular metabolic viability following exposure to the extracts. Therefore, chemical radical-scavenging activity cannot be directly interpreted as cellular antioxidant protection, nor does it preclude cytotoxic effects in cultured cells. Accordingly, the present findings should be interpreted as evidence of chemical radical-scavenging capacity, together with concentration-dependent cytotoxic/antiproliferative activity, in the tested cancer-derived cell models. Determination of whether these effects are selective toward neoplastic cells, mediated by oxidative stress, or associated with functional apoptosis requires further investigation using appropriate non-neoplastic controls and mechanistic assays. First, plant materials from three individual plants from a single population were pooled before extraction. Thus, the three replicates represent independent extraction replicates from one composite biological sample rather than independent plant-level biological replicates. Therefore, statistical comparisons between leaf and stem extracts describe reproducibility within the pooled sample and cannot establish generalizable organ-specific differences for the species. The limited number of independent extraction replicates (*n* = 3) also restricts the strength of statistical inference. Second, plant material was collected from a single location and during one season, limiting geographic and seasonal generalizability. Third, GC–MS profiling may under-represent non-volatile compounds, and most detected compounds were tentatively identified using spectral libraries; confirmation using authentic standards and complementary techniques such as high-performance liquid chromatography (HPLC), liquid chromatography–mass spectrometry (LC–MS/MS), and nuclear magnetic resonance (NMR) is required. Glucosinolates and isothiocyanates were not comprehensively characterized, and chemical standardization using quantified marker compounds was not performed. Fourth, DPPH and ABTS^+^ assays measure chemical radical-scavenging capacity under cell-free conditions and therefore do not establish cellular or in vivo antioxidant effects. Future studies should evaluate reactive oxygen species (ROS), malondialdehyde (MDA), and antioxidant enzyme activities. Fifth, the absence of non-tumor human cells prevented assessment of tumor selectivity and calculation of a selectivity index. Sixth, the α-glucosidase assay used *Saccharomyces cerevisiae* enzyme, which may not fully represent mammalian or human intestinal α-glucosidase activity. Seventh, all pharmacological assays were performed in vitro, limiting extrapolation to in vivo efficacy because bioavailability, metabolism, pharmacokinetics, and toxicity were not assessed. Eighth, apoptosis-associated effects were evaluated primarily at the mRNA level. Functional assays, including Annexin V/propidium iodide (PI) staining, caspase activity, cleaved caspases and PARP, protein-level Bax/Bcl-2 assessment, and mitochondrial membrane-potential analysis, were not performed. In addition, the RT-qPCR assays were not fully validated according to Minimum Information for Publication of Quantitative Real-Time PCR Experiments (MIQE) recommendations, including comprehensive assessment of RNA quality, amplification efficiency, primer specificity, appropriate controls, and reference-gene stability. Therefore, the gene-expression findings, particularly caspase-3 expression in MCF-7 cells, should be considered preliminary.

Finally, potential matrix interference from extract color or turbidity may affect spectrophotometric assays, although extract-specific controls were incorporated for several assays. Overall, these limitations indicate that the findings should be considered preliminary in vitro evidence and require validation using independently sampled plants, comprehensive chemical characterization, appropriate biological controls, functional mechanistic assays, and in vivo studies.

## 5. Conclusions

This study characterized and compared the phytochemical profiles and in vitro bioactivity patterns of methanolic SILE and SISE from *S. irio*. L. GC–MS analysis revealed distinct organ-specific chemical profiles, with SILE characterized by relatively higher proportions of cycloartane triterpenoids and α-linolenic acid, whereas SISE contained relatively higher proportions of phytosterols, tocopherols, and pyranone derivatives. SISE showed greater chemical radical-scavenging capacity in the DPPH and ABTS^+^ assays, whereas SILE exhibited stronger antibacterial activity against several of the tested bacterial strains, based on the ZoI measurements and MIC/MBC endpoint determinations, and greater in vitro α-amylase-/α-glucosidase-inhibitory activities. Both extracts also produced concentration-dependent reductions in cellular viability in the MCF-7 and HepG2 cell models. Changes in the expression of caspase-3, caspase-8, caspase-9, Bax, Bcl-2, and Bcl-xL suggest the involvement of apoptosis-associated transcriptional responses; however, these findings do not establish functional apoptosis, tumor selectivity, or an anticancer effect. Similarly, the DPPH and ABTS^+^ results demonstrate chemical radical-scavenging capacity rather than cellular or in vivo antioxidant activity, while in vitro α-amylase/α-glucosidase inhibition represents preliminary in vitro enzyme-inhibitory activity rather than evidence of an antihyperglycemic effect.

Overall, the findings provide preliminary evidence of organ-specific phytochemical composition and multiple in vitro bioactivities of *S. irio* L. extracts. The observed differences between leaves and stems suggest organ-specific variation in their phytochemical profiles and biological activities. However, these findings do not validate the ethnopharmacological use of *S. irio* L. or establish therapeutic efficacy. Further studies should incorporate chemical standardization using quantified marker compounds, bioassay-guided fractionation, comprehensive LC–MS/MS and complementary phytochemical analyses, appropriate non-neoplastic cell controls, functional apoptosis assays, mammalian intestinal enzyme systems, and cellular and in vivo models. Additional toxicological, pharmacokinetic, mechanistic, and in vivo efficacy studies are also required before considering potential clinical applications.

## Figures and Tables

**Figure 1:**
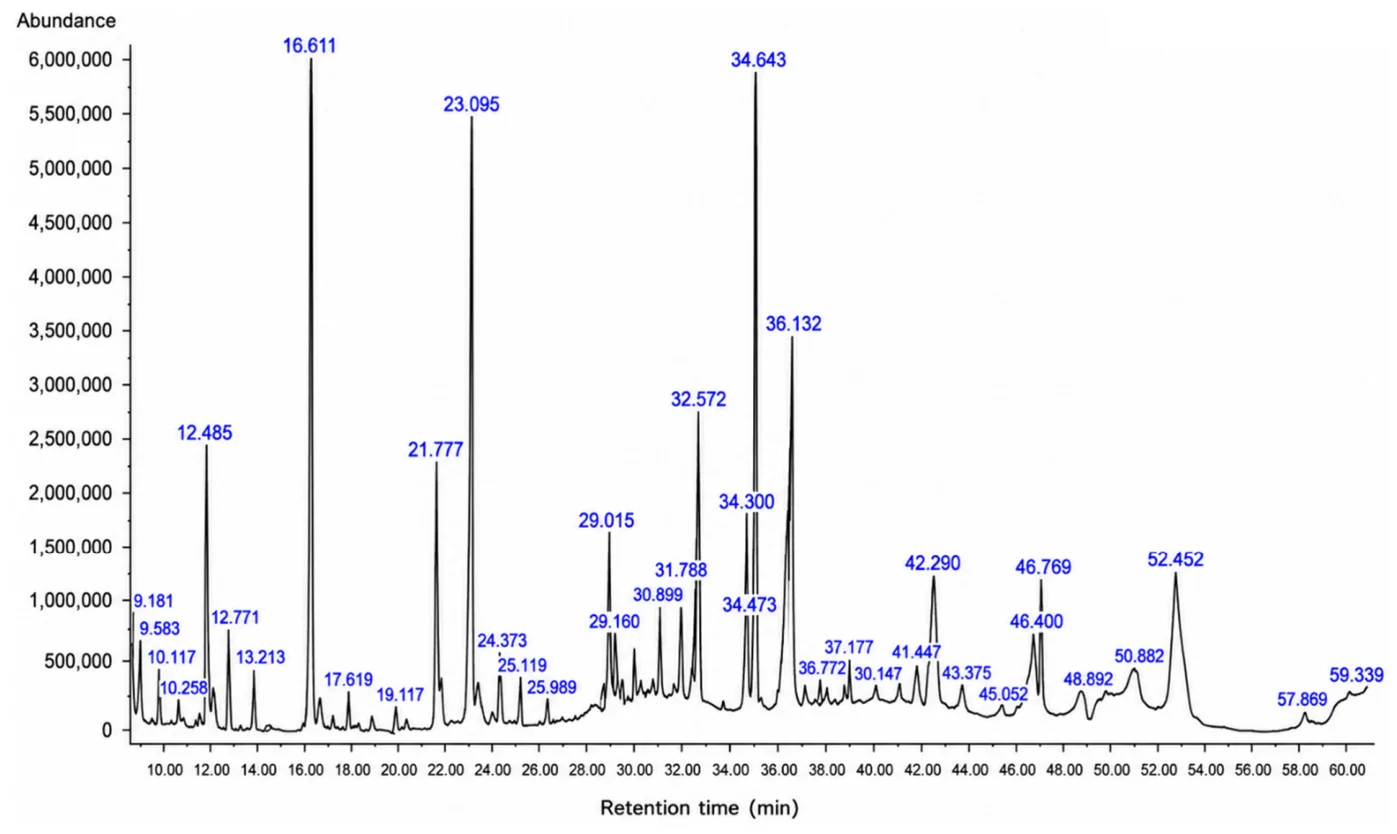
GC–MS chromatogram of SILE showing the distribution of putatively identified compounds. Each spectral peak corresponds to a specific phytochemical constituent, with the dominant peak representing the major compound in the extract.

**Figure 2:**
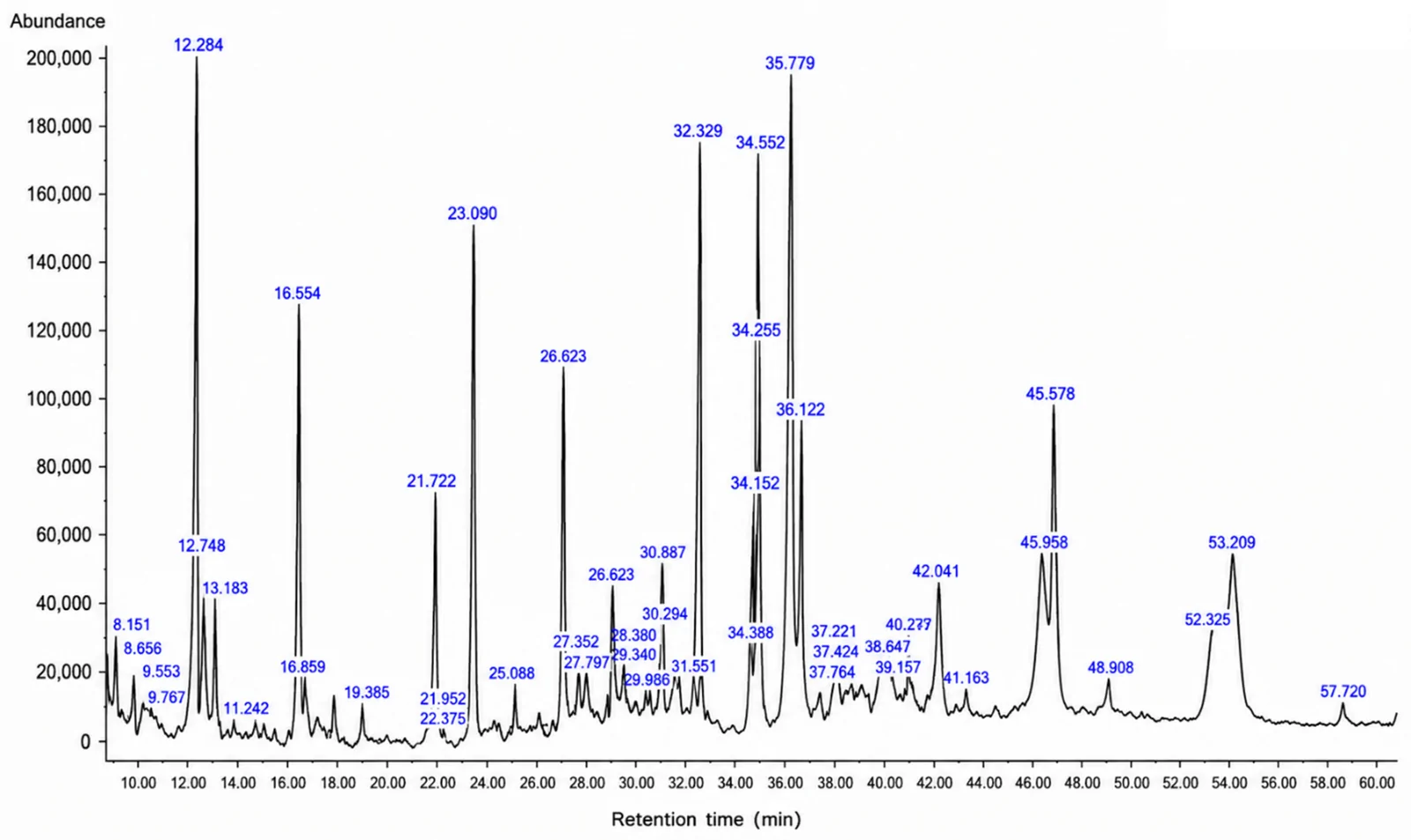
GC–MS chromatogram of *SISE* showing the distribution of putatively identified compounds. Each spectral peak corresponds to a specific phytochemical constituent, with the dominant peak representing the major compound in the extract.

**Figure 3:**
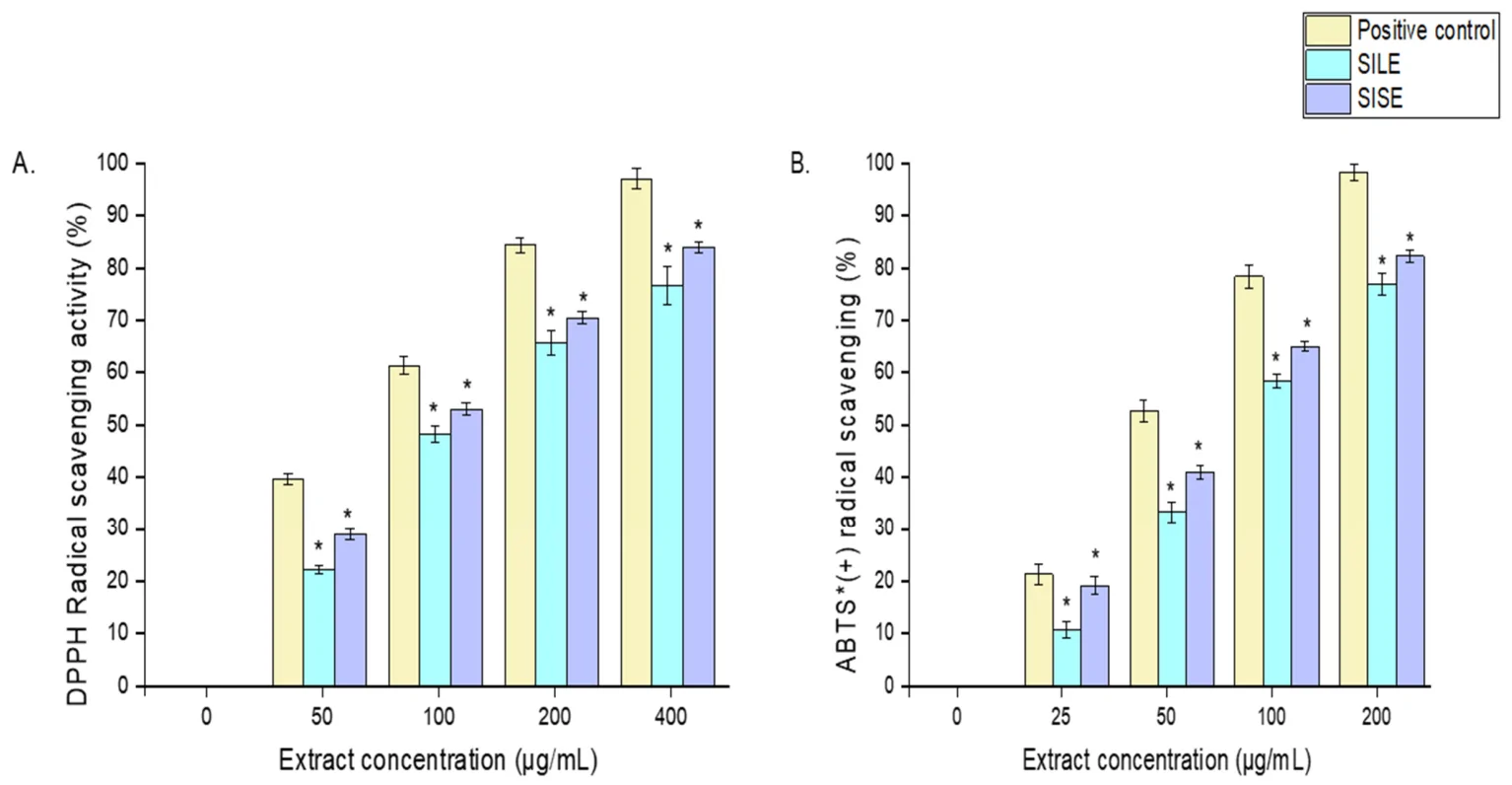
DPPH (**A**) and ABTS^+^ (**B**) radical-scavenging capacities of SILE and SISE. Extracts were tested at 0 (methanol, vehicle control), 100, 200, and 400 μg/mL for DPPH and 0 (methanol, vehicle control), 50, 100, and 200 μg/mL for ABTS^+^. Ascorbic acid was included as a positive reference control. Data are presented as mean ± SD (*n* = 3 independent extraction/experimental replicates from the same pooled plant-organ material). Where technical triplicates were performed, measurements were averaged before statistical analysis. Statistical comparisons were performed by two-way ANOVA followed by Tukey’s multiple-comparison test. * *p* < 0.05 versus positive reference control (Ascorbic acid). (+), radical cation. IC_50_ values were determined by nonlinear regression and are reported with 95% CIs where estimable. Concentration–response curves were fitted using a 4PL nonlinear regression model. For DPPH, R^2^ = 1.000, 1.000, and 0.99997 for ascorbic acid, SILE, and SISE, respectively; for ABTS^+^, R^2^ = 1.000 for all fitted curves.

**Figure 4:**
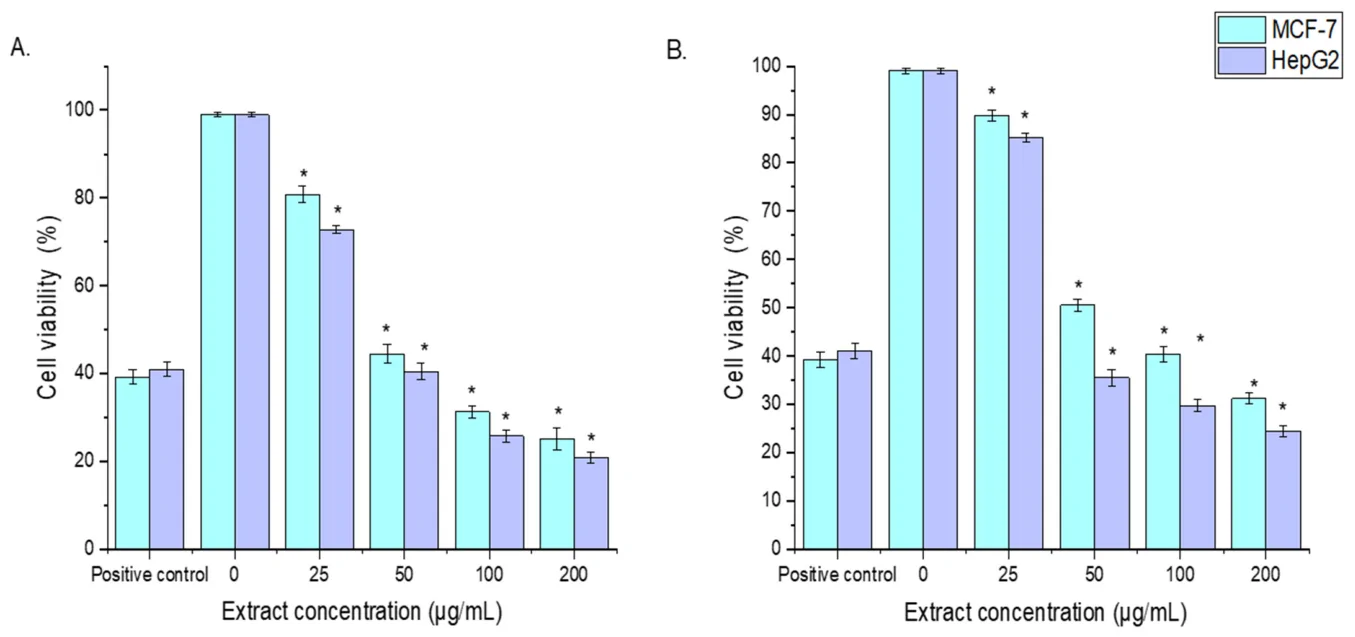
In vitro cytotoxic/antiproliferative effects of SILE (**A**) and SISE (**B**) on MCF-7 and HepG2 cancer cells following 24 h exposure, as determined by the MTT assay. Extracts were tested at 0 (0.01% DMSO, vehicle control), 25, 50, 100, and 200 μg/mL. Cisplatin (30 μg/mL) was included as a positive reference control. Data are presented as mean ± SD (*n* = 3 independent extraction/experimental replicates from the same pooled plant-organ material). Statistical comparisons were performed by three-way ANOVA followed by Tukey’s multiple-comparison test. * *p* < 0.05 versus the positive reference control (cisplatin). Concentration–response curves were fitted using a 4PL nonlinear regression model. R^2^ values were 0.9998, 1.0000, 0.9949, and 0.9966 for SILE/MCF-7, SILE/HepG2, SISE/MCF-7, and SISE/HepG2, respectively. IC_50_ values were determined by nonlinear regression and are reported with their corresponding 95% CIs.

**Figure 5:**
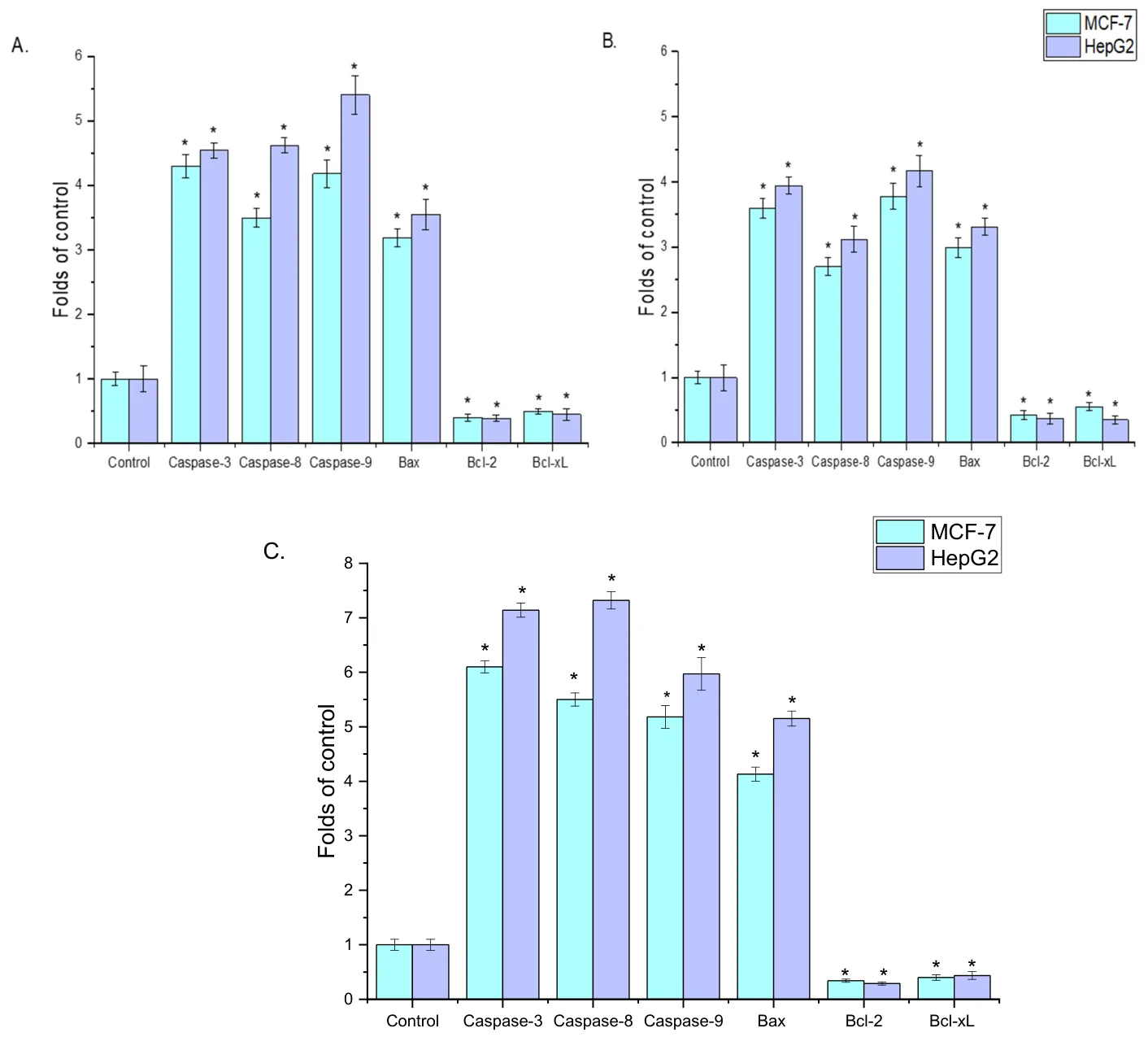
Effect of SILE (**A**), SISE (**B**), and cisplatin (30 μg/mL) (**C**) on apoptosis-associated gene expression in MCF-7 and HepG2 cells. Relative mRNA expression levels of caspase-3, caspase-8, caspase-9, Bax, Bcl-2, and Bcl-xL were calculated using the 2^−ΔΔCt^ method and are presented as fold changes relative to 0.01% DMSO vehicle-treated controls. Data are presented as mean ± SD (*n* = 3 independent biological replicates). Technical PCR replicates were averaged before statistical analysis. Statistical comparisons were performed on ΔCt values using a two-sided unpaired Student’s *t*-test. * *p* < 0.05 versus the corresponding 0.01% DMSO vehicle control. The RT-qPCR findings represent preliminary apoptosis-associated transcriptional responses and do not establish functional apoptosis. The CASP3 result in MCF-7 cells should therefore be interpreted particularly cautiously.

**Figure 6:**
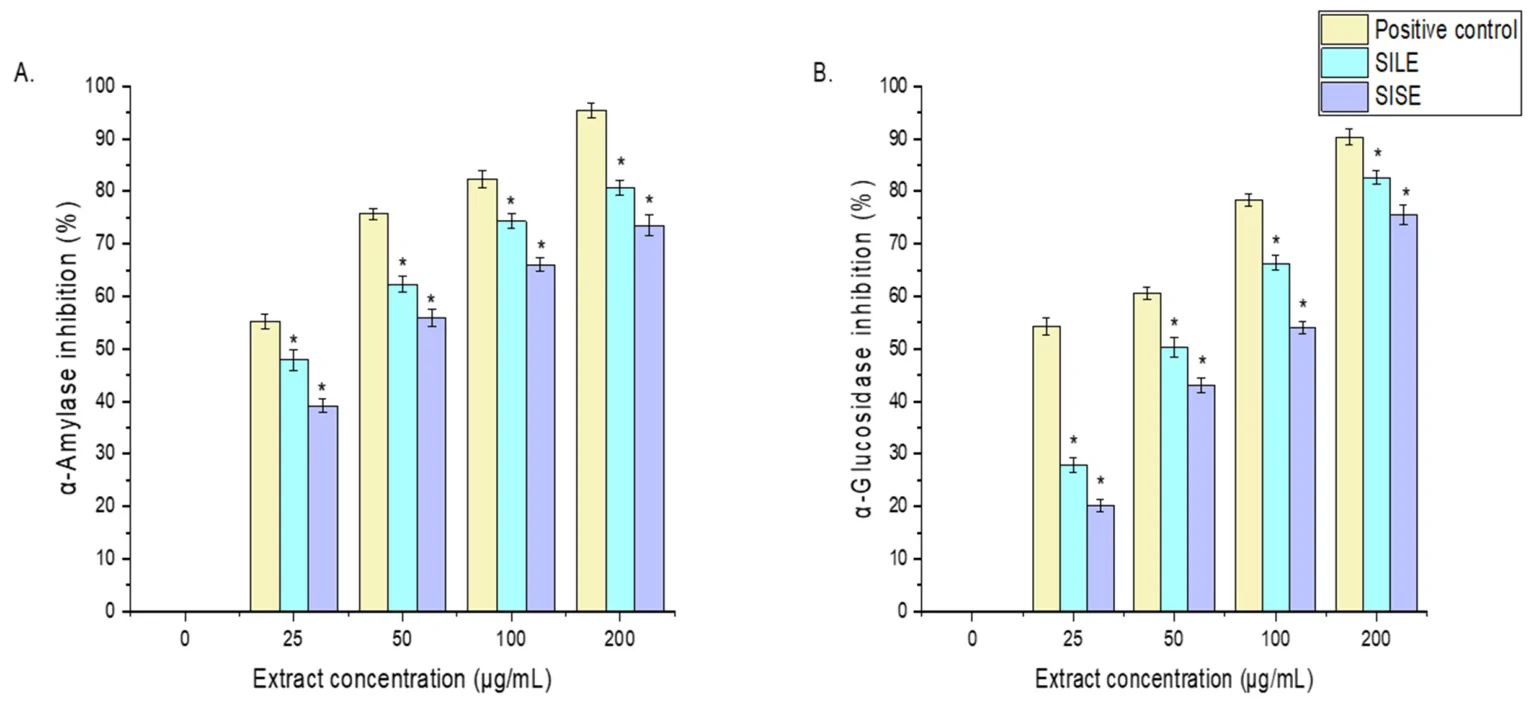
In vitro α-amylase (**A**) and α-glucosidase (**B**) inhibitory activities of SILE and SISE. Extracts were tested at 0 (0.01% DMSO, vehicle control), 25, 50, 100, and 200 μg/mL. Acarbose was used as the positive control. Data are presented as mean ± SD (*n* = 3 independent extraction/experimental replicates from the same pooled plant-organ material). Where technical triplicates were performed, measurements were averaged before statistical analysis. Statistical comparisons were performed by two-way ANOVA followed by Tukey’s multiple-comparison test. * *p* < 0.05 versus the positive reference control (acarbose). Concentration–response curves were fitted using a 4PL nonlinear regression model. For α-amylase, R^2^ = 0.9790, 1.0000, and 0.9997 for acarbose, SILE, and SISE, respectively; for α-glucosidase, R^2^ = 1.0000, 0.9988, and 0.9847, respectively. IC_50_ values were calculated by nonlinear regression and are reported with 95% CIs.

**Table 1:** Summary of plant material, extraction conditions, and experimental samples used in the study.

Plant Material	Extract Code	Material Used	Solvent	Solid-to-Solvent Ratio	Extraction Time	Extraction Temperature	Replicates
Leaves	SILE	10 g dried powdered leaves	100% methanol	1:10 (*w*/*v*)	48 h	25 ± 2 °C	3 independent extraction replicates
Stems	SISE	10 g dried powdered stems	100% methanol	1:10 (*w*/*v*)	48 h	25 ± 2 °C	3 independent extraction replicates

**Table 2:** The primer sequences of various genes involved in apoptosis and anti-apoptotic genes.

Gene Name	Primer’s Sequence (5′–3′)	Reference
*Caspase-3*	F: 5′-GCTGGATGCCGTCTAGAGTC-3′	[22].
	R: 5′-ATGTGTGGATGATGCTGCCA-3′	
*Caspase-8*	F: 5′-AGAAGAGGGTCATCCTGGGAGA-3′	[23].
	R: 5′- TCAGGACTTCCTTCAAGGCTGC-3′	
*Caspase-9*	F: 5′- ATTGCACAGCACGTTCACAC-3′	[22].
	R: 5′-TATCCCATCCCAGGAAGGCA-3′	
*Bax*	F: 5′-GAGCTAGGGTCAGAGGGTCA-3′	[22].
	R: 5′-CCCCGATTCATCTACCCTGC-3′	
*Bcl-2*	F: 5′-ACCTACCCAGCCTCCGTTAT-3′	[22].
	R: 5′-GAACTGGGGGAGGATTGTGG-3′	
*Bcl-XL*	F: 5′-CAGAGCTTTGAACAGGTAG-3′	[24].
	R: 5′-GCTCTCGGGTGCTGTATTG-3′	
*GAPDH*	F: 5′- CGGAGTCAACGGATTTGGTC -3′	[25].
	R: 5′- AGCCTTCTCCATGGTCGTGA -3′	

**Table 3:** GC–MS compounds in SILE.

Peak	Rt	Area	Area%	Name	MF	Mw	CAS No.	Classification	Similarity Score	ID Level
1	8.181	60,272,955	0.99	*cis*-p-Mentha-1(7),8-dien-2-ol	C_10_H_16_O	152	13544-59-7	Monoterpenoid	92	2
2	8.597	4,342,096	0.07	4-methyl-cyclohex-2-en-1-ol	C_7_H_12_O	112	Not found	Terpenoid derivative	85	3
3	9.583	24,528,193	0.4	2-acetyl-3,4,5,6-tetrahydropyridine	C_7_H_11_NO	125	27300-27-2	Pyridine alkaloid	88	3
4	9.706	5,739,657	0.09	3-hydroxy-4-methylbenzaldehyde	C_8_H_8_O_2_	136	57295-30-4	Phenolic aldehyde	91	2
5	9.883	3,554,172	0.06	isovanillin	C_8_H_8_O_3_	152	621-59-0	Phenolic aldehyde	93	2
6	10.171	13,803,741	0.23	furaneol	C_6_H_8_O_3_	128	3658-77-3	Furanone	94	2
7	10.258	3,874,829	0.06	niobe oil	C_8_H_8_O_2_	136	93-58-3	Aromatic ester	92	2
8	11.938	7,989,437	0.13	tropinone	C_8_H_13_NO	139	532-24-1	Tropane alkaloid	90	2
9	12.019	9,329,456	0.15	6-nonenal, 3,7-dimethyl-	C_11_H_20_O	168	Not found	Monoterpenoid	86	3
10	12.485	356,226,626	5.87	4H-pyran-4-one, 2,3-dihydro-3,5-dihydroxy-6-methyl-	C_6_H_8_O_4_	144	28564-83-2	Pyranone	95	2
11	12.653	9,557,308	0.16	γ-terpineol, dihydro-	C_10_H_20_O	156	498-81-7	Monoterpenoid	91	2
12	12.771	28,918,083	0.48	levomenthol	C_10_H_20_O	156	2216-51-5	Monoterpenoid	96	2
13	13.213	20,370,457	0.34	ethyl 3-hexenoate	C_8_H_14_O_2_	142	2396-83-0	Fatty acid ester	90	2
14	13.654	14,492,099	0.24	cyclohexanecarboxylic acid, 4-methyl-	C_8_H_14_O_2_	142	4331-54-8	Cycloalkane carboxylic acid	88	3
15	14.907	19,552,648	0.32	o-tolualdehyde	C_8_H_8_O	120	529-20-4	Aromatic aldehyde	92	2
16	14.998	11,881,880	0.2	2-cyclohexen-1-one, 4-hydroxy-3-methyl-6-(1-methylethyl)-, trans-	C_10_H_16_O_2_	168	141978-04-3	Terpenoid derivative	87	3
17	16.611	308,464,750	5.09	2-methoxy-4-vinylphenol	C_9_H_10_O_2_	150	7786-61-0	Phenolic	94	2
18	16.897	15,636,065	0.26	3,5-heptadienal, 2-ethylidene-6-methyl-	C_10_H_14_O	150	99172-18-6	Monoterpenoid	89	3
19	17.337	23,542,696	0.39	Benzene, 2-(2-butenyl)-1,3,5-trimethyl-	C_13_H_18_	174	63435-25-6	Aromatic hydrocarbon	86	3
20	17.529	8,518,589	0.14	naphthalene, 1,2-dihydro-1,1,6-trimethyl-	C_13_H_16_	172	30364-38-6	Dihydronaphthalene	88	3
21	17.619	17,546,498	0.29	eugenol	C_10_H_12_O_2_	164	97-53-0	Phenylpropanoid	95	2
22	18.161	17,358,629	0.29	p-anisic acid, methyl ester	C_9_H_10_O_3_	166	121-98-2	Aromatic ester	91	2
23	18.502	6,574,997	0.11	naphthalene, 1,2-dihydro-3,5,8-trimethyl-	C_13_H_16_	172	30316-18-8	Dihydronaphthalene	87	3
24	19.026	9,015,144	0.15	1,1,5-trimethyl-1,2-dihydronaphthalene	C_13_H_16_	172	Not found	Dihydronaphthalene	85	2
25	19.115	16,735,283	0.28	2-hydroxy-6-methyl-3-cyclohexen-1-carboxylic acid	C_8_H_12_O_3_	156	Not found	Hydroxy acid	84	3
26	19.417	15,370,929	0.25	myristoleic acid	C_14_H_26_O_2_	226	544-64-9	Unsaturated fatty acid	90	2
27	20.342	7,209,844	0.12	(−)-pinane-3-carboxylic acid	C_11_H_18_O_2_	182	58096-27-8	Terpenoid acid	86	3
28	20.5	8,007,278	0.13	isopinocarveol	C_10_H_16_O	152	6712-79-4	Monoterpenoid	87	3
29	20.742	12,704,043	0.21	3-hydroxy-α-ionene	C_13_H_20_O_2_	208	Not found	Norisoprenoid	85	3
30	20.895	7,449,235	0.12	undecylenic acid	C_11_H_20_O_2_	184	112-38-9	Unsaturated fatty acid	89	3
31	21.777	157,578,449	2.6	p-menthane-1,2,3-triol	C_10_H_20_O_3_	188	104153-60-8	Monoterpenoid triol	89	3
32	21.952	42,302,210	0.7	alloaromadendrene epoxide	C_15_H_24_O	220	85760-81-2	Sesquiterpene epoxide	88	3
33	22.864	19,989,928	0.33	acetoveratrone	C_10_H_12_O_3_	180	1131-62-0	Aromatic ketone	90	2
34	23.095	280,526,885	4.62	3′,5′-dimethoxyacetophenone	C_10_H_12_O_3_	180	39151-19-4	Aromatic ketone	93	2
35	23.282	18,616,336	0.31	megastigmatrienone	C_13_H_18_O	190	13215-88-8	Norisoprenoid	89	3
36	23.448	14,874,428	0.25	4-(2,4,4-trimethyl-cyclohexa-1,5-dienyl)-but-3-en-2-one	C_13_H_18_O	190	Not found	Norisoprenoid	86	3
37	23.571	15,993,250	0.26	8-ketocopaenal	C_15_H_20_O_2_	232	136396-58-2	Sesquiterpenoid aldehyde	85	3
38	23.729	29,020,120	0.48	2H-2,4a-methanonaphthalene, 1,3,4,5,6,7-hexahydro-1,1,5,5-tetramethyl-, (*2S*)-	C_15_H_24_	204	1135-66-6	Sesquiterpene	88	3
39	24.046	14,916,763	0.25	ylangenal	C_15_H_22_O	218	41610-68-8	Sesquiterpenoid aldehyde	85	3
40	24.373	35,358,024	0.58	aristolene	C_15_H_24_	204	6831-16-9	Sesquiterpene	86	3
41	24.862	9,470,930	0.16	longiverbenone	C_15_H_22_O	218	64180-68-3	Sesquiterpenoid ketone	87	3
42	25.119	22,667,090	0.37	benzaldehyde, 2-hydroxy-4-methoxy-3,6-dimethyl-	C_10_H_12_O_3_	180	34883-15-3	Phenolic aldehyde	85	3
43	25.989	12,891,129	0.21	4-hydroxy-β-ionone	C_13_H_20_O_2_	208	14398-34-6	Norisoprenoid	88	3
44	26.939	5,327,019	0.09	1-naphthalenecarboxylic acid, methyl ester	C_12_H_10_O_2_	186	2459-24-7	Naphthoate ester	90	2
45	27.466	3,241,048	0.05	7-oxabicyclo [4.1.0]heptane, 1-(1,3-dimethyl-1,3-butadienyl)-2,2,6-trimethyl-, (*E*)-	C_15_H_24_O	220	89128-14-3	Epoxy terpenoid	84	3
46	27.649	10,062,490	0.17	γ-gurjunenepoxide-(1)	C_15_H_24_O	220	Not found	Sesquiterpene	86	3
47	27.797	12,442,732	0.21	isolongifolol	C_15_H_26_O	222	1139-17-9	Sesquiterpenoid alcohol	87	3
48	27.965	25,428,716	0.42	α-santonin	C_15_H_18_O_3_	246	481-06-1	Sesquiterpene lactone	91	2
49	28.092	13,784,976	0.23	caryophylla-4(12),8(13)-dien-5α-ol	C_15_H_24_O	220	19431-79-9	Sesquiterpenoid alcohol	85	3
50	28.858	18,924,024	0.31	aromandendrene	C_15_H_24_	204	489-39-4	Sesquiterpene	88	3
51	29.015	57,741,493	0.95	exo-ketoborneol	C_10_H_16_O_2_	168	12001-40-0	Monoterpenoid ketone	86	3
52	29.16	29,052,712	0.48	*trans*-2-dodecen-1-ol	C_12_H_24_O	184	69064-37-5	Fatty alcohol	88	3
53	29.395	41,657,649	0.69	*E*-3-Pentadecen-2-ol	C_15_H_30_O	226	Not found	Fatty alcohol	85	3
54	29.531	30,345,133	0.5	*trans*-Sinapaldehyde	C_11_H_12_O_4_	208	4206-58-0	Phenylpropanoid aldehyde	92	2
55	29.91	19,920,084	0.33	neophytadiene	C_20_H_38_	278	504-96-1	Diterpene	93	2
56	30.054	7,644,953	0.13	elemicin	C_12_H_16_O_3_	208	487-11-6	Phenylpropanoid	91	2
57	30.245	32,913,242	0.54	8-isopropenyl-1,3,3,7-tetramethyl-bicyclo [5.1.0]oct-5-en-2-one	C_15_H_22_O	218	Not found	Sesquiterpenoid ketone	86	3
58	30.554	7,966,075	0.13	β-cyclocostunolide	C_15_H_20_O_2_	232	2221-82-1	Sesquiterpene lactone	90	2
59	31.506	7,710,987	0.13	1,3,12-nonadecatriene	C_19_H_34_	262	646052-22-4	Hydrocarbon	85	3
60	31.788	85,869,148	1.42	9,12,15-octadecatrienal	C_18_H_30_O	262	26537-71-3	Polyunsaturated aldehyde	91	2
61	32.572	377,369,981	6.22	n-hexadecanoic acid	C_16_H_32_O_2_	256	57-10-3	Saturated fatty acid	98	2
62	33.465	4,599,588	0.08	sclarene	C_20_H_32_	272	511-02-4	Diterpene	89	3
63	34.172	30,042,676	0.5	manool	C_20_H_34_O	290	596-85-0	Diterpenoid alcohol	90	2
64	34.473	37,996,037	0.63	sinapic acid methyl ester	C_12_H_14_O_5_	238	20733-94-2	Phenylpropanoid ester	93	2
65	34.643	300,286,012	4.95	phytol	C_20_H_40_O	296	150-86-7	Diterpenoid	96	2
66	36.132	950,451,222	15.67	α-linolenic acid	C_18_H_30_O_2_	278	463-40-1	Polyunsaturated fatty acid	97	2
67	37.498	13,656,797	0.23	11,13-dimethyl-12-tetradecen-1-ol acetate	C_18_H_34_O_2_	282	Not found	Fatty alcohol acetate	84	3
68	37.79	17,222,869	0.28	2-hexadecanol	C_16_H_34_O	242	14852-31-4	Fatty alcohol	88	3
69	38.352	14,772,128	0.24	*cis*-10-nonadecenoic acid	C_19_H_36_O_2_	296	73033-09-7	Unsaturated fatty acid	90	2
70	38.888	7,363,902	0.12	δ-dodecalactone	C_12_H_22_O_2_	198	713-95-1	Lactone	89	3
71	39.687	14,033,294	0.23	arachidic acid	C_20_H_40_O_2_	312	506-30-9	Saturated fatty acid	92	2
72	40.783	33,819,559	0.56	1-naphthalenepropanol, α-ethenyldecahydro-2-hydroxy-α,2,5,5,8a-pentamethyl-, [*1R*-[1α(R*),2β,4aβ,8aα]]-	C_20_H_36_O_2_	308	515-03-7	Diterpenoid	86	3
73	41.449	23,265,074	—	*cis*-13-Eicosenoic acid	C_20_H_38_O_2_	310	17735-94-3	Unsaturated fatty acid	90	2
74	41.667	50,823,460	0.84	kolavelool	C_20_H_34_O	290	19941-81-2	Diterpenoid alcohol	88	3
75	42.29	190,384,570	3.14	2-monopalmitin	C_19_H_38_O_4_	330	23470-00-0	Monoacylglycerol	89	3
76	43.375	33,586,415	0.55	docosanoic acid	C_22_H_44_O_2_	340	112-85-6	Saturated fatty acid	91	2
77	46.4	205,886,456	3.39	monolinolenin	C_21_H_36_O_4_	352	18465-99-1	Monoacylglycerol	89	3
78	48.577	76,399,157	1.26	squalene	C_30_H_50_	410	111-02-4	Triterpene	94	2
79	49.445	49,308,152	0.81	lupeol	C_30_H_50_O	426	545-47-1	Triterpenoid	92	2
80	49.543	15,157,256	0.25	2-[5-(2,2-dimethyl-6-methylene-cyclohexyl)-3-methyl-pent-2-enyl]-[1,4]benzoquinone	C_21_H_28_O_2_	312	Not found	Benzoquinone terpenoid	84	3
81	50.882	225,883,092	3.72	oxidosqualene	C_30_H_50_O	426	54910-48-4	Triterpenoid	88	3
82	59.339	2,662,315	0.04	γ-tocopherol (vitamin E)	C_28_H_48_O_2_	416	54-28-4	Tocopherol	95	2

**Table 4:** GC–MS compounds in *SISE*.

Peak	Rt	Area	Area%	Name	MF	MW	CAS No	Class	Similarity Score	ID Level
1	8.151	11,047,502	0.43	1-octen-3-ol	C_8_H_16_O	128	3391-86-4	Fatty alcohol	91	2
2	8.589	1,682,433	0.07	4-methyl-cyclohex-2-en-1-ol	C_7_H_12_O	112	Not found	Cyclic alcohol	84	3
3	8.732	4,120,191	0.16	2-cyclopenten-1-one, 3,4,4-trimethyl-	C_8_H_12_O	124	30434-65-2	Cyclic ketone	87	3
4	9.518	17,487,124	0.68	2-acetyl-3,4,5,6-tetrahydropyridine	C_7_H_11_NO	125	27300-27-2	Pyridine alkaloid	88	3
5	9.722	16,150,715	0.63	3-hydroxy-4-methylbenzaldehyde	C_8_H_8_O_2_	136	57295-30-4	Phenolic aldehyde	91	2
6	10.007	27,639,858	1.08	furaneol	C_6_H_8_O_3_	128	3658-77-3	Furanone	94	2
7	10.276	19,253,390	0.75	benzoic acid, methyl ester	C_8_H_8_O_2_	136	93-58-3	Aromatic ester	92	2
8	11.124	3,894,962	0.15	1,2-benzenediol, 4-methyl-	C_7_H_8_O_2_	124	452-86-8	Catechol	90	2
9	11.907	1,984,956	0.08	tropinone	C_8_H_13_NO	139	532-24-1	Tropane alkaloid	90	2
10	12.284	207,203,524	8.09	4H-Pyran-4-one, 2,3-dihydro-3,5-dihydroxy-6-methyl-	C_6_H_8_O_4_	144	28564-83-2	Pyranone	95	2
11	12.748	16,763,630	0.65	Levomenthol	C_10_H_20_O	156	2216-51-5	Monoterpenoid	96	2
12	12.914	3,451,116	0.13	2(*5H*)-Furanone, 4-methyl-5-(2-methyl-2-propenyl)-	C_9_H_12_O_2_	152	89902-24-9	Lactone	86	3
13	13.183	13,338,382	0.52	decanal	C_10_H_20_O	156	112-31-2	Fatty aldehyde	89	3
14	13.316	2,452,539	0.1	3-hexenoic acid, ethyl ester	C_8_H_14_O_2_	142	2396-83-0	Fatty acid ester	90	2
15	13.541	10,643,721	0.42	cyclohexanecarboxylic acid, 4-methyl-	C8H14O2	142	4331-54-8	Cycloalkane carboxylic acid	88	3
16	14.977	4,536,679	0.18	o-tolualdehyde	C_8_H_8_O	120	529-20-4	Aromatic aldehyde	92	2
17	15.068	4,444,177	0.17	2-cyclohexen-1-one, 4-hydroxy-3-methyl-6-(1-methylethyl)-, trans-	C_10_H_16_O_2_	168	141978-04-3	Terpenoid ketone	87	3
18	15.569	1,885,027	0.07	1,11-dodecadiene	C_12_H_22_	166	5876-87-9	Alkadiene	85	3
19	15.713	2,999,038	0.12	8-Hydroxymenthol	C_10_H_20_O_2_	172	3564-98-5	Monoterpenoid diol	86	3
20	16.554	75,380,317	2.94	2-methoxy-4-vinylphenol	C_9_H_10_O_2_	150	7786-61-0	Phenolic	94	2
21	16.868	17,882,843	0.7	3,5-heptadienal, 2-ethylidene-6-methyl-	C10H_14_O	150	99172-18-6	Monoterpenoid	89	3
22	18.051	2,594,553	0.1	vanillol	C_8_H_10_O_3_	154	498-00-0	Phenolic alcohol	91	2
23	18.145	9,430,527	0.37	p-anisic acid	C_9_H_10_O_3_	166	121-98-2	Aromatic ester	92	2
24	18.396	2,207,676	0.09	p-Anisylacetone	C_10_H_12_O_2_	164	122-84-9	Aromatic ketone	88	3
25	18.488	3,983,830	0.16	naphthalene, 1,2-dihydro-3,5,8-trimethyl-	C_13_H_16_	172	30316-18-8	Dihydronaphthalene	87	3
26	19.091	5,551,608	0.22	1,1,5-Trimethyl-1,2-dihydronaphthalene	C_13_H_16_	172	Not found	Dihydronaphthalene	85	3
27	19.386	6,212,354	0.24	trans-2-Undecenoic acid	C_11_H_20_O_2_	184	15790-94-0	Unsaturated fatty acid	90	2
28	20.325	2,007,589	0.08	(−)-Pinane-3-carboxylic acid	C_11_H_18_O_2_	182	58096-27-8	Terpenoid acid	86	3
29	20.722	2,931,689	0.11	3-hydroxy-α-ionene	C_13_H_20_O_2_	208	Not found	Norisoprenoid	85	3
30	20.871	2,425,600	0.09	isomethyl-α-ionol	C_14_H_24_O	208	51595-91-6	Terpenoid alcohol	87	3
31	21.722	51,099,540	2	p-menthane-1,2,3-triol	C_10_H_20_O_3_	188	104153-60-8	Monoterpenoid triol	89	3
32	21.933	6,408,715	0.25	alloaromadendrene oxide	C_15_H_24_O	220	85760-81-2	Sesquiterpene epoxide	88	3
33	22.79	4,273,039	0.17	acetoveratrone	C_10_H_12_O_3_	180	1131-62-0	Aromatic ketone	90	2
34	23.009	62,446,564	2.44	3′,5′-dimethoxyacetophenone	C_10_H_12_O_3_	180	39151-19-4	Aromatic ketone	93	2
35	23.363	2,434,740	0.1	megastigmatrienone	C_13_H_18_O	190	13215-88-8	Norisoprenoid	89	3
36	23.51	4,335,802	0.17	4-(2,4,4-trimethyl-cyclohexa-1,5-dienyl)-but-3-en-2-one	C_13_H_18_O	190	Not found	Norisoprenoid	86	3
37	23.635	2,343,890	0.09	8-ketocopaenal	C_15_H_20_O_2_	232	136396-58-2	Sesquiterpenoid aldehyde	85	3
38	23.71	4,914,621	0.19	2H-2,4a-methanonaphthalene, 1,3,4,5,6,7-hexahydro-1,1,5,5-tetramethyl-, (2*S*)-	C_15_H_24_	204	1135-66-6	Sesquiterpene	88	3
39	24.336	3,476,425	0.14	Cyclopenta [1,3]cyclopropa [1,2]cyclohepten-3(3aH)-one, 1,2,3b,6,7,8-hexahydro-6,6-dimethyl-	C_13_H_18_O	190	Not found	Sesquiterpenoid	84	3
40	24.828	1,559,091	0.06	longiverbenone	C_15_H_22_O	218	64180-68-3	Sesquiterpenoid ketone	87	3
41	25.088	5,672,522	0.22	*trans*-2-dodecenoic acid	C_12_H_22_O_2_	198	32466-54-9	Unsaturated fatty acid	89	3
42	25.936	6,961,954	0.27	terpinyl formate	C_11_H_18_O_2_	182	2153-26-6	Monoterpenoid ester	88	3
43	26.387	2,711,571	0.11	ascaridole epoxide	C_10_H_16_O_3_	184	135760-25-7	Monoterpenoid peroxide	86	3
44	26.571	5,900,356	0.23	c-β-elemenone	C_15_H_22_O	218	20303-60-0	Sesquiterpenoid ketone	88	3
45	26.649	2,875,964	0.11	caryophyllene oxide	C_15_H_24_O	220	1139-30-6	Sesquiterpene epoxide	91	2
46	26.788	3,785,700	0.15	longipinocarveol, trans-	C_15_H_24_O	220	Not found	Sesquiterpenoid alcohol	85	3
47	26.923	51,300,918	2	1-naphthalenecarboxylic acid, methyl ester	C_12_H_10_O_2_	186	2459-24-7	Naphthoate ester	90	2
48	27.186	6,230,418	0.24	caryophyllene	C_15_H_24_	204	87-44-5	Sesquiterpene	92	2
49	27.594	38,231,984	1.49	γ-Gurjunenepoxide	C_15_H_24_O	220	Not found	Sesquiterpene epoxide	86	3
50	27.879	13,710,240	0.54	myristic acid	C_14_H_28_O_2_	228	544-63-8	Saturated fatty acid	93	2
51	28.07	4,154,980	0.16	n-Tridecan-1-ol	C_13_H_28_O	200	112-70-9	Fatty alcohol	88	3
52	28.212	16,549,027	0.65	linalyl acetate	C_12_H_20_O_2_	196	115-95-7	Monoterpenoid ester	91	2
53	28.75	12,654,075	0.49	2-octen-1-ol, 3,7-dimethyl-, isobutyrate, (*Z*)-	C_14_H_26_O_2_	226	Not found	Monoterpenoid ester	85	3
54	28.983	20,082,048	0.78	dodeca-1,6-dien-12-ol, 6,10-dimethyl-	C_14_H_26_O	210	Not found	Diterpenoid alcohol	84	3
55	29.134	11,720,425	0.46	*epi-*α-cadinol	C_15_H_26_O	222	1-11-5937	Sesquiterpenoid	89	3
56	29.34	20,869,117	0.81	ferulic acid methyl ester	C_11_H_12_O_4_	208	2309-07-1	Phenylpropanoid ester	93	2
57	29.505	11,629,196	0.45	piperiton epoxid	C_10_H_16_O_2_	168	5286-38-4	Monoterpenoid epoxide	87	3
58	29.882	6,805,558	0.27	3,7,11,15-tetramethyl-2-hexadecen-1-ol	C_20_H_40_O	296	150-86-7	Diterpenoid	90	2
59	29.985	4,898,263	0.19	neophytadiene	C_20_H_38_	278	504-96-1	Diterpene	93	2
60	30.214	9,610,807	0.38	geranyl isovalerate	C_15_H_26_O_2_	238	109-20-6	Monoterpenoid ester	89	3
61	30.52	8,608,576	0.34	β-cyclocostunolide	C_15_H_20_O_2_	232	2221-82-1	Sesquiterpene lactone	90	2
62	30.758	6,311,162	0.25	13-octadecenal, (*Z*)-	C_18_H_34_O	266	58594-45-9	Fatty aldehyde	87	3
63	31.475	3,331,027	0.13	*trans*-sinapaldehyde	C_11_H_12_O_4_	208	4206-58-0	Phenylpropanoid aldehyde	92	2
64	31.605	13,818,537	0.54	7,11-hexadecadienal	C_16_H_28_O	236	96883-53-3	Unsaturated aldehyde	87	3
65	32.329	166,454,145	6.5	n-hexadecanoic acid	C_16_H_32_O_2_	256	57-10-3	Saturated fatty acid	98	2
66	32.447	8,168,835	0.32	*trans*-2-Hexadecenoic acid	C_16_H_30_O_2_	254	929-79-3	Unsaturated fatty acid	89	3
67	34.398	13,336,982	0.52	sinapic acid methyl ester	C_12_H_14_O_5_	238	20733-94-2	Phenylpropanoid ester	93	2
68	34.552	71,179,643	2.78	Phytol	C_20_H_40_O	296	150-86-7	Diterpenoid	96	2
69	35.779	310,439,960	12.12	α-linolenic acid	C_18_H_30_O_2_	278	463-40-1	Polyunsaturated fatty acid	97	2
70	36.122	94,773,356	3.7	stearic acid	C_18_H_36_O_2_	284	57-11-4	Saturated fatty acid	94	2
71	37.442	10,471,876	0.41	11,13-dimethyl-12-tetradecen-1-ol acetate	C_18_H_34_O_2_	282	Not found	Fatty alcohol acetate	84	3
72	37.613	9,327,885	0.36	elaidic acid	C_18_H_34_O_2_	282	112-79-8	Trans-fatty acid	89	3
73	37.721	14,400,659	0.56	2-hexadecanol	C_16_H_34_O	242	14852-31-4	Fatty alcohol	88	3
74	37.851	18,727,954	0.73	1-nonadecene	C_19_H_38_	266	18435-45-5	Alkene	86	3
75	38.294	20,339,934	0.79	*cis*-10-Nonadecenoic acid	C_19_H_36_O_2_	296	73033-09-7	Unsaturated fatty acid	90	2
76	39.587	56,082,077	2.19	vitamin E	C_29_H_50_O_2_	430	59-02-9	Tocopherol	95	2
77	40.707	25,416,789	0.99	1-naphthalenepropanol, α-ethenyldecahydro-2-hydroxy-α,2,5,5,8a-pentamethyl-, [*1R*-[1α(R*),2β,4aβ,8aα]]-	C_20_H_36_O_2_	308	515-03-7	Diterpenoid	86	3
78	41.37	7,784,608	0.3	erucic acid	C_22_H_42_O_2_	338	112-86-7	Unsaturated fatty acid	90	2
79	42.041	63,983,396	2.5	palmitic acid β-monoglyceride	C_19_H_38_O_4_	330	23470-00-0	Monoacylglycerol	88	3
80	43.15	7,797,700	0.3	docosanoic acid	C_22_H_44_O_2_	340	112-85-6	Saturated fatty acid	91	2
81	44.708	4,249,141	0.17	9,10-secocholesta-5,7,10(19)-triene-3,24,25-triol, (3β,*5Z*,*7E*)-	C_27_H_44_O_3_	416	40013-87-4	Secosteroid	85	3
82	45.958	150,435,385	5.87	1-linolenoylglycerol	C_21_H_36_O_4_	352	18465-99-1	Monoacylglycerol	89	3
83	48.408	10,936,015	0.43	squalene	C_30_H_50_	410	111-02-4	Triterpene	94	2
84	52.365	52,966,129	2.07	γ-sitosterol	C_29_H_50_O	414	83-47-6	Phytosterol	93	2
85	53.209	183,606,233	7.17	β-sitosterol	C_29_H_50_O	414	83-46-5	Phytosterol	96	2

**Table 5:** ZoI (mm), MIC, and MBC values of SILE.

Bacterium/Dilution	Chloramphenicol 25 µg/mL	800 μg/mL	400 μg/mL	200 μg/mL	100 μg/mL	MIC μg/mL	MBC μg/mL
*S. aureus*	23 ± 2.15	20 ± 2.57 *	18 ± 0.98 *	16 ± 1.53 *	13 ± 1.15 *	6.25	12.50
*E. faecalis*	24 ± 1.49	21 ± 2.11 *	17 ± 2.81 *	15 ± 0.66 *	13 ± 1.16 *	6.25	12.50
*B. subtilis*	20 ± 1.49	19 ± 1.97 *	16 ± 0.78 *	14 ± 1.45 *	12 ± 0.24 *	12.50	25.00
*E. coli*	21 ± 1.73	18 ± 0.96 *	17 ± 0.86 *	14 ± 1.19 *	11 ± 1.82 *	50.00	100.00
*K. pneumoniae*	20 ± 1.48	17 ± 1.98 *	15 ± 0.75 *	13 ± 1.51 *	11 ± 1.19 *	25.00	50.00
*P. aeruginosa*	22 ± 1.95	17 ± 1.35 *	14 ± 0.64 *	14 ± 1.56 *	10 ± 0.77 *	25.00	50.00

Note: Zone of inhibition (ZoI, mm), minimum inhibitory concentration (MIC, µg/mL), and minimum bactericidal concentration (MBC, µg/mL) of SILE against six bacterial strains. SILE was tested at 100, 200, 400, and 800 µg/mL, with 0.01% DMSO used as the vehicle control. Chloramphenicol (25 µg/mL) was included for reference comparison and was not used as the basis for determining treatment effects. Data are presented as mean ± SD (*n* = 3 independent extraction/experimental replicates from the same pooled plant-organ material). Where technical triplicates were performed, measurements were averaged before statistical analysis. ZoI data were analyzed by three-way ANOVA followed by Tukey’s multiple-comparison test, with treatment comparisons made against the corresponding positive control (25 µg/mL chloramphenicol), as indicated by * (*p* < 0.05). MIC and MBC were determined by two-fold broth microdilution, and the reported values represent the lowest endpoints obtained across three independent determinations.

**Table 6:** The ZoI (mm), MIC (μg/mL), and MBC (μg/mL) values of SISE.

Bacterium/Dilution	Chloramphenicol, 25 µg/mL	800 μg/mL	400 μg/mL	200 μg/mL	100 μg/mL	MIC μg/mL	MBC μg/mL
*S. aureus*	23 ± 2.15	18 ± 1.85	14 ± 1.52 *	12 ± 1.25 *	10 ± 1.96 *	25.00	50.00
*E. faecalis*	24 ± 1.49	17 ± 2.15	15 ± 1.51 *	13 ± 1.42 *	11 ± 0.42 *	12.50	25.00
*B. subtilis*	20 ± 1.49	18 ± 1.43	14 ± 2.13 *	11 ± 0.95 *	10 ± 0.72 *	12.50	25.00
*E. coli*	21 ± 1.73	16 ± 0.63	13 ± 1.16 *	10 ± 0.29 *	9 ± 1.42 *	50.00	100.00
*K. pneumoniae*	20 ± 1.48	15 ± 1.42	14 ± 1.41 *	9 ± 0.37 *	7 ± 1.55	25.00	50.00
*P. aeruginosa*	22 ± 1.95	16 ± 1.25	12 ± 1.37 *	9 ± 1.84 *	8 ± 1.25 *	50.00	100.00

Note: Zone of inhibition (ZoI, mm), minimum inhibitory concentration (MIC, µg/mL), and minimum bactericidal concentration (MBC, µg/mL) of SISE against six bacterial strains. SISE was tested at 100, 200, 400, and 800 µg/mL, with 0.01% DMSO used as the vehicle control. Chloramphenicol (25 µg/mL) was included for reference comparison and was not used as the basis for determining treatment effects. Data are presented as mean ± SD (*n* = 3 independent extraction/experimental replicates from the same pooled plant-organ material). Where technical triplicates were performed, measurements were averaged before statistical analysis. ZoI data were analyzed by three-way ANOVA followed by Tukey’s multiple-comparison test, with treatment comparisons made against the corresponding positive control (25 µg/mL chloramphenicol), as indicated by * (*p* < 0.05). MIC and MBC were determined by two-fold broth microdilution, and the reported values represent the lowest endpoints obtained across three independent determinations.

## Data Availability

The original contributions presented in this study are included in the article. Further inquiries can be directed to the corresponding authors.

## Abbreviations

The following abbreviations are used in this manuscript:

GC–MS	Gas Chromatography–Mass Spectrometry
FCRC	Folin–Ciocalteu reducing capacity
TFC	Total Flavonoid Content
DPPH	2,2-Diphenyl-1-picrylhydrazyl
ABTS	2,2′-Azino-bis(3-ethylbenzothiazoline-6-sulfonic acid)
MIC	Minimum Inhibitory Concentration
MBC	Minimum Bactericidal Concentration
